# Re-envisaging child protection contacts as an early prevention opportunity to support child development and well-being: an Australian data linkage study

**DOI:** 10.1136/jech-2024-223006

**Published:** 2025-02-26

**Authors:** Kathleen Falster, Rhiannon Megan Pilkington, Tasnia Ahmed, Alicia Montgomerie, Mark Hanly, BJ Newton, Marni Brownell, Ben Edwards, Raghu Lingam, Anthony Shakeshaft, Michelle Cretikos, Jessica Stewart, Katherine Hawkins, Kitty McClean, John W Lynch

**Affiliations:** 1 School of Population Health, University of New South Wales, Sydney, New South Wales, Australia; 2 School of Public Health, The University of Adelaide, Adelaide, South Australia, Australia; 3 Centre for Big Data Research in Health, University of New South Wales, Sydney, New South Wales, Australia; 4 Social Policy Research Centre, University of New South Wales, Sydney, New South Wales, Australia; 5 Manitoba Centre for Health Policy, Department of Community Health Sciences, University of Manitoba, Winnipeg, Manitoba, Canada; 6 Centre for Social Policy Research, Australian National University, Canberra, Australian Capital Territory, Australia; 7 School of Women’s and Children’s Health, University of New South Wales, Sydney, New South Wales, Australia; 8 Poche Centre for Indigenous Health Services Research, The University of Queensland, Brisbane, Queensland, Australia; 9 National Drug and Alcohol Centre, University of New South Wales, Sydney, New South Wales, Australia; 10 Centre for Epidemiology and Evidence, NSW Ministry of Health, Sydney, New South Wales, Australia; 11 Family and Community Services Insights, Analysis and Research, NSW Department of Communities and Justice, Sydney, New South Wales, Australia; 12 Child and Family Support, SA Department of Human Services, Adelaide, South Australia, Australia; 13 Quality and Practice, SA Government Department of Child Protection, Adelaide, South Australia, Australia; 14 Bristol Medical School, Population Health Sciences, University of Bristol, Bristol, UK

**Keywords:** CHILD HEALTH, EPIDEMIOLOGY, Life course epidemiology, PREVENTION, PUBLIC HEALTH

## Abstract

**Objectives:**

To quantify developmental vulnerability at age 5 by child protection contacts before school in two Australian states.

**Methods:**

All children with birth, child protection and/or 2009, 2012, 2015 and 2018 Australian Early Development Census (AEDC) data in New South Wales (NSW) and South Australia (SA) were grouped according to child protection contact before school: no contact, child protection reports, screened-in reports, investigations, substantiations and out-of-home care (OOHC). The outcome was developmental vulnerability on ≥1 AEDC domains or medically diagnosed conditions with support needs at school entry.

**Results:**

56 650 (14.2%) NSW children and 12 617 (15.6%) SA children had ≥1 child protection contact before school. Developmental vulnerability on ≥1 domains or medically diagnosed conditions was lowest in the no child protection group (NSW, 21–22%; SA, 24–25%), with progressively higher risk in the child protection report (NSW, 35%; SA, 41–46%) through to the OOHC (NSW, 50–54%; SA, 59–66%) groups in all AEDC years. Developmental risk was higher among children aged <2 years at first contact and those with more reports. Children with only one child protection report before school had approximately 65% higher developmental risk than the no child protection group in both states.

**Conclusions:**

A single child protection report before school was an early indicator of higher developmental risk at age 5, with higher developmental risks among children with earlier, more serious and frequent child protection contacts. Beyond child safety screening, child protection reports represent an opportunity to mobilise early health and social support for children with developmental support needs.

**WHAT IS ALREADY KNOWN ON THIS TOPIC**

- Numerous child protection system reviews have highlighted the need to shift from crisis-driven responses to the prevention of maltreatment and its health and developmental consequences for children in the child protection system.
- The design and delivery of such prevention initiatives require data infrastructure and evidence on the opportunities to respond to early indicators of developmental risk, such as child and family contacts with health, child protection and social services, in whole populations of children.
- Policy and service delivery planning requires the use of data that truly reflects the whole population potentially eligible for supportive services for early child development.

**WHAT THIS STUDY ADDS**

- Children with just a single child protection report before school entry had about 65% higher risk of teacher-reported developmental vulnerability on ≥1 domains or medically diagnosed conditions at age 5, in whole child populations in two Australian jurisdictions with different child protection legislation.
- Children with more serious, frequent or earlier child protection contacts had progressively higher risks of developmental vulnerability on ≥1 domains or medically diagnosed conditions at age 5 in whole child populations in two Australian jurisdictions and consistently across four developmental census years.

**HOW THIS STUDY MIGHT AFFECT RESEARCH, PRACTICE OR POLICY**

- Beyond their vital function to screen and respond to imminent child safety and risk, child protection reports represent an interagency opportunity to mobilise referrals to health system-led screening, assessment and early, non-judgemental support focused on child health and development in at-risk families during early childhood and health and education supports from school entry.
- This study highlights how actionable targets for public health prevention—whole population numbers and characteristics of children with developmental support needs—can be generated using information captured in government administrative data systems reflecting real-life service interactions, outcomes and prevention opportunities across agencies.

## Introduction

 Children who experience abuse and neglect have worse health, developmental and long-term life outcomes than other children.^1^,^3^ Numerous reports have highlighted the crisis-oriented child protection system and the urgent need to invest in the prevention of child maltreatment and its consequences.^4^,^9^ The planning and implementation of primary and secondary prevention measures such as targeted supportive interventions relies on population-level data infrastructure and intelligence on early indicators of a child’s future risk of poor outcomes that services, if properly resourced, can respond to. If early contact with the child protection system reliably indicates the population burden of future developmental risk for children, such descriptive epidemiological evidence can inform resourcing and mobilisation of health system-led screening, assessment and early, non-judgemental support focused on child health and development in at-risk families.

There are prevention opportunities at every stage of the child protection system. The first indicator of risk is an initial report of safety concerns from health professionals and others to the child protection helpline. These reports may then be screened-in, investigated, substantiated and possibly lead to a court order to remove the child to out-of-home care (OOHC). Five previous publications have used whole population data on child protection and teacher-reported developmental vulnerability at school entry; all excluded children with medically diagnosed conditions with substantial support needs.^210^,^13^ One Canadian report showed developmental vulnerability among more than half of children in OOHC, 44% of children with other child protection contacts and one-quarter of children with no child protection contact.^2^ Another Canadian study used similar data from Manitoba, comparing developmental vulnerability among children in OOHC versus all other children, thus combining children with other child protection contacts and those who had no contact.^13^ Three Australian studies^10^,^12^ all estimated associations between different categorisations of child protection contact on developmental vulnerability at school entry. The modelling process involved adjustment for various covariates, such as socioeconomic and perinatal health characteristics. Covariate adjustment creates an ‘imaginary world’^14^ where the estimated quantity (eg, an odds ratio) represents the association between an exposure and outcome as if all individuals had the same level of the covariate. Of course, in the real world they do not. Such estimates are not quantities that are actionable by policymakers. Paraphrasing Rose *et al*,—relative risk is for researchers, decision-making requires absolutes.^15^

The purpose of the current study was to generate actionable targets for public health prevention, using best practice descriptive epidemiology,^16 17^ by quantifying the burden of teacher-reported developmental vulnerability on ≥1 domains and medically diagnosed conditions at school entry across all levels of child protection contact in whole child populations. We do this in two Australian states with different child protection legislation and practices and across four whole population censuses of child development at age 5 in 2009, 2012, 2015 and 2018.

## Methods

### Study design

Cohort study using record linkage of population data in two jurisdictions (online supplemental eFigure 1).

### Data sources and data linkage

The New South Wales (NSW) Centre for Health Record Linkage and SANT DataLink linked the data for the NSW Child E-Cohort Project and BEBOLD (Better Evidence, Better Outcomes, Linked Data) in South Australia (SA), respectively (false positive linkages, 0.4–0.5%).^18 19^ We used data from birth registrations, perinatal statistics, hospital birth records (NSW only), public school enrolments (NSW only) and child protection in NSW and SA and the 2009, 2012, 2015 and 2018 Australian Early Development Census (AEDC) in this paper.^20^

### Study population

We assembled open cohorts to represent the target populations, including all children who were eligible for support, any time from pregnancy to school entry, from health, child protection and/or education services. Most children entered the cohorts at birth (86%), based on perinatal, birth registration and/or hospital records. Others entered the cohorts after birth, based on child protection and/or AEDC records with a residential area and/or school in NSW or SA.

We first assembled records from all four AEDC cycles for children with birth, child protection and/or AEDC records between birth and ≤31 January in their first year of school (NSW, N=401 598; SA, N=81 639). Study population flow charts including the numbers of children with birth, child protection and/or AEDC records are detailed in online supplemental figures S2–S3. After exclusions, there were 398 702 NSW children and 80 731 SA children in our main analysis sample with a valid AEDC DV1 (i.e. developmentally vulnerable on ≥1 domains) summary indicator or medically diagnosed conditions.

### Child protection population and contacts

We defined the child protection population in each state using child protection contacts ≤31 January in the year children started school (Australian school year starts late January/early February). Although NSW and SA have different child protection legislation, policies, procedures and terminology, agencies in both states have intake processes for child protection reports, which the system may screen in as meeting definitions of harm or risk of harm, investigate, substantiate and may result in removing the child to OOHC.^21^ We summarised this progression through the child protection systems into mutually exclusive groups as ‘the most serious type of contact’ (online supplemental table S1), and we examined the age at first contact (derived using the date of the first report, or OOHC placement for the <0.1% with no prior report) and the number of child protection reports before school.

### Developmental outcomes at school entry

Outcomes were ascertained from the AEDC, which is a triennial, nationwide census of child development among Australian children in their first year of full-time school (aged 4.5–6, average of 5 years).^20^ The AEDC collects teacher-reported data on five domains: (1) physical health and well-being; (2) social competence; (3) emotional maturity; (4) language and cognitive skills (school-based); and (5) communication skills and general knowledge.^20^ The AEDC has high population coverage (96.0–99.9% in NSW; 87.8–96.9% in SA).^22^,^25^

#### Teacher-reported developmental vulnerability

For each developmental domain, children receive a score between 0 and 10, where lower scores are more developmentally vulnerable. In 2009, cut-off scores were established for three AEDC domain indicator categories: (1) ‘Developmentally vulnerable’ included scores <10th percentile; (2) ‘Developmentally at risk’ included scores ≥10th and ≤25th percentile; (3) ‘Developmentally on track’ included scores >25th percentile. The 2009 cut-off scores have remained the same across all AEDC cycles and provide a reference point to compare more recent AEDC results.^20^ We examined the number and combinations of developmentally vulnerable domains and the AEDC DV1 summary indicator (ie, developmentally vulnerable on ≥1 domains). Children with medically diagnosed conditions with AEDC-defined ‘special needs’ (online supplemental table S2) are not included in the AEDC domain indicators because of the already identified substantial developmental needs of this group.^25 26^

#### Medically diagnosed conditions with support needs

We used the AEDC indicator for medically diagnosed conditions with ‘special needs’ (henceforth ‘support needs’) as an additional outcome because our goal was to quantify the population burden of all developmental support needs.

### Socio-demographic characteristics of children and families

The following socio-demographic characteristics were used to describe the study population: child’s sex, maternal country of birth, Aboriginal and/or Torres Strait Islander (First Nations) child/parent, maternal marital/partner status at child’s birth, private health insurance/patient at child’s birth, English second language (child), maternal education, highest parent/carer occupation, area-level disadvantage, remoteness of area of residence, AEDC year and whether the child repeated their first year of school.

### Statistical analysis

For each jurisdiction, we described the socio-demographic characteristics stratified by the most serious type of child protection contact. We calculated the incidence proportion (henceforth ‘risk’) of developmental vulnerability on ≥1 domains or medically diagnosed conditions among the total at-risk population in NSW and SA from birth to school age, stratified by the: most serious child protection contact before school in each AEDC year; age at first child protection contact (combined AEDC years); number of child protection reports before school (combined AEDC years); and combinations of the type, timing and frequency of child protection contacts before school. Among children with valid AEDC domains (analysis sample 2), we quantified the number and percentage of children with the most common types and combinations of domain developmental vulnerability, stratified by the most serious child protection contact, with cell sizes <5 suppressed.

#### Sensitivity analysis

We investigated the impact of incomplete data on child protection reports and investigations in NSW in 2003/2004 on the ascertainment of child protection contacts for the 2009 NSW AEDC cohort.

### Ethics approval

Ethics approval was obtained from multiple ethics committees in NSW and SA (see Acknowledgements).

## Results

### Child protection populations

The population with child protection contact before school consisted of 56 650 (14.2%) NSW children and 12 617 (15.6%) SA children (online supplemental eTable 3). The mutually exclusive most serious child protection contact groups included: child protection reports, 9986 (2.5%) NSW and 4121 (5.1%) SA children; screened-in child protection reports, 30 848 (7.7%) NSW and 4336 (5.4%) SA children; investigations, 2283 (0.6%) NSW and 1672 (2.1%) SA children; substantiations, 8161 (2.0%) NSW and 1544 (1.9%) SA children; and OOHC, 5372 (1.4%) NSW and 944 (1.2%) SA children (online supplemental eTable 3).

Sensitivity analyses of re-reporting and investigations in the 2012–2018 AEDC cohorts suggest <1% to 2% point under-ascertainment of reports and investigations by school age in the 2009 AEDC cohort (online supplemental eTable 4).

### Demographic profile of children and families, by most serious child protection contact

Child’s sex and age at AEDC were similarly distributed across the most serious child protection contact groups in both jurisdictions (online supplemental eTable 5). More common characteristics among children with more serious child protection contacts included: parent/s aged <20 years at child’ birth; mother not married/partnered at child’s birth; Australian-born mother; First Nations child/parent; English first language (child); highest maternal education level <year 12 or not reported/available; no parent in the labour force; public patient/hospital (child’s birth); more disadvantaged area of residence; and regional area of residence (online supplemental eTable 5).

### Risk of developmental vulnerability on ≥1 domains or medically diagnosed conditions, by child protection contact

#### Most serious child protection contact before school

**Figure 1:**
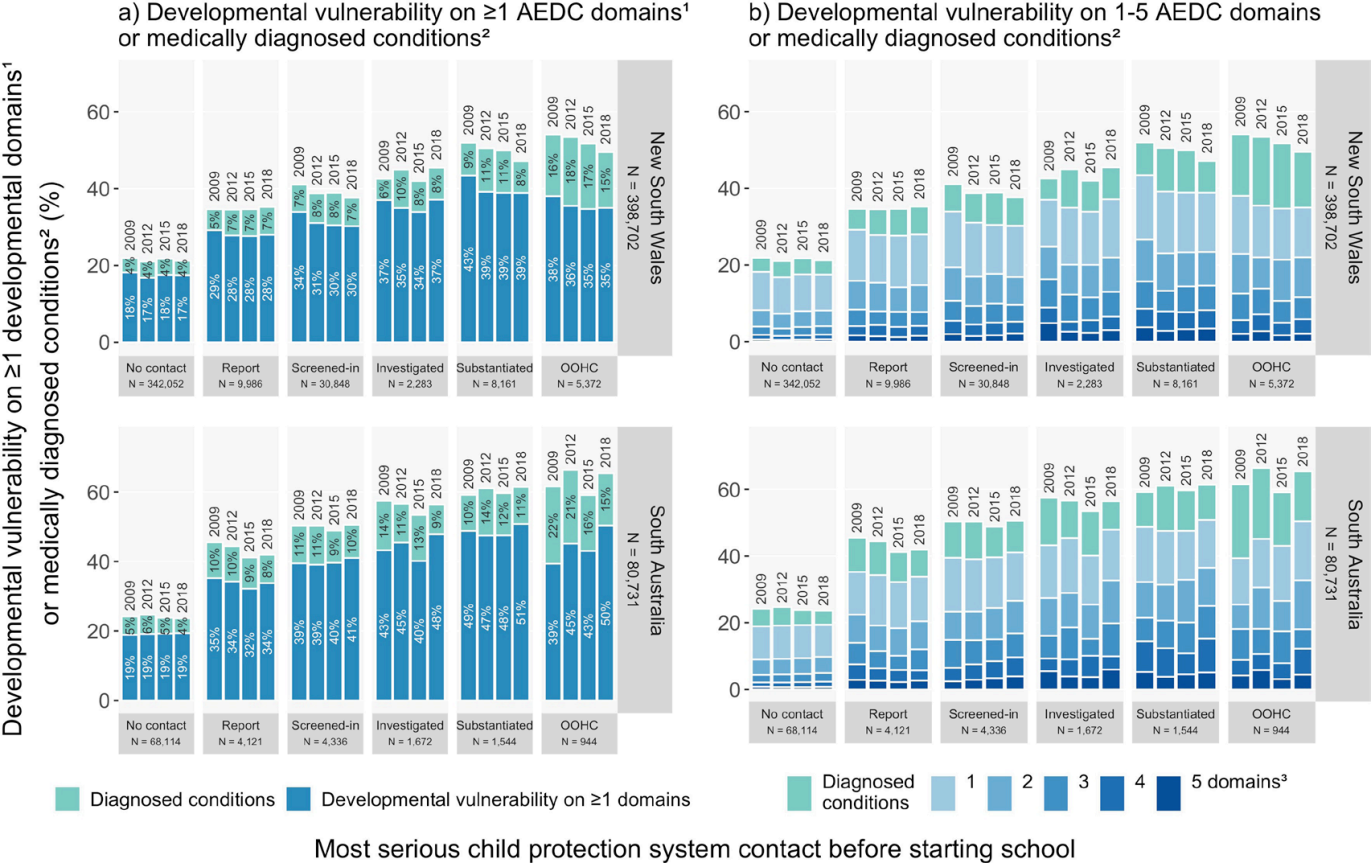
The risk of developmental vulnerability on: (**a**) ≥1 AEDC domains^1^, or (**b**) 1–5 AEDC domains, or medically diagnosed conditions^2^, by most serious child protection contact before school, among New South Wales and South Australian children in their first year of full-time school in 2009, 2012, 2015 or 2018 (see data in online supplemental eTable 8 and 9). Report, child protection report; screened-in, child protection reports that were screened by the system to meet a threshold of risk/harm in each jurisdiction; investigated, child protection services investigation of alleged maltreatment; substantiated, maltreatment that was confirmed by child protection services. The OOHC data includes placements related to care and protection orders, as well as voluntary care, emergency care and respite placements for a short period. (1) The AEDC DV1 summary indicator was used for the outcome ‘developmental vulnerability on ≥1 AEDC domains’; (2) medically diagnosed conditions with already identified substantial developmental support needs, as listed in online supplemental eTable 2; (3) The figure 1b legend shows the gradient of blue colours that indicate the per cent of children who were developmentally vulnerable on 1, 2, 3, 4 or 5 AEDC domains. AEDC, Australian Early Developmental Census; DV1, developmentally vulnerable on ≥1 AEDC domains; OOHC, out-of-home care placement.

Figure 1a shows that, in both jurisdictions, the risk of developmental vulnerability on ≥1 domains was lowest in the no child protection group (NSW, 17–18%; SA, 19%), with progressively higher risk in the child protection report (NSW, 28–29%; SA, 32–35%) through to the OOHC (NSW, 35–38%; SA, 39–50%) groups in all AEDC years. The risk of medically diagnosed conditions was also lowest in the no child protection group (NSW, 4%; SA, 4–6%) and highest in the OOHC group (NSW, 15–18%; SA, 15–22%) in all AEDC years.

Figure 1b shows that, in both jurisdictions, the risk of developmental vulnerability in one domain was most common, with progressively lower risk of developmental vulnerability on two to five domains, across all child protection contact groups and AEDC years. Although risk estimates varied across AEDC years, the risk of developmental vulnerability on any number of domains was higher among children with versus without child protection contact. For example, the risk of developmental vulnerability on one domain was lowest in the no child protection contact group (NSW, 9.3–10.0%; SA, 9.6–10.0%) and highest in the OOHC group (NSW, 12.9–15.1%; SA, 13.9–17.6%) in all AEDC years (figure 1b). The risk of developmental vulnerability on five domains was 0.4–0.8% in the no child protection group and 1.6–5.8% in the OOHC group across census years in both states.

#### Age at first child protection contact

**Figure 2:**
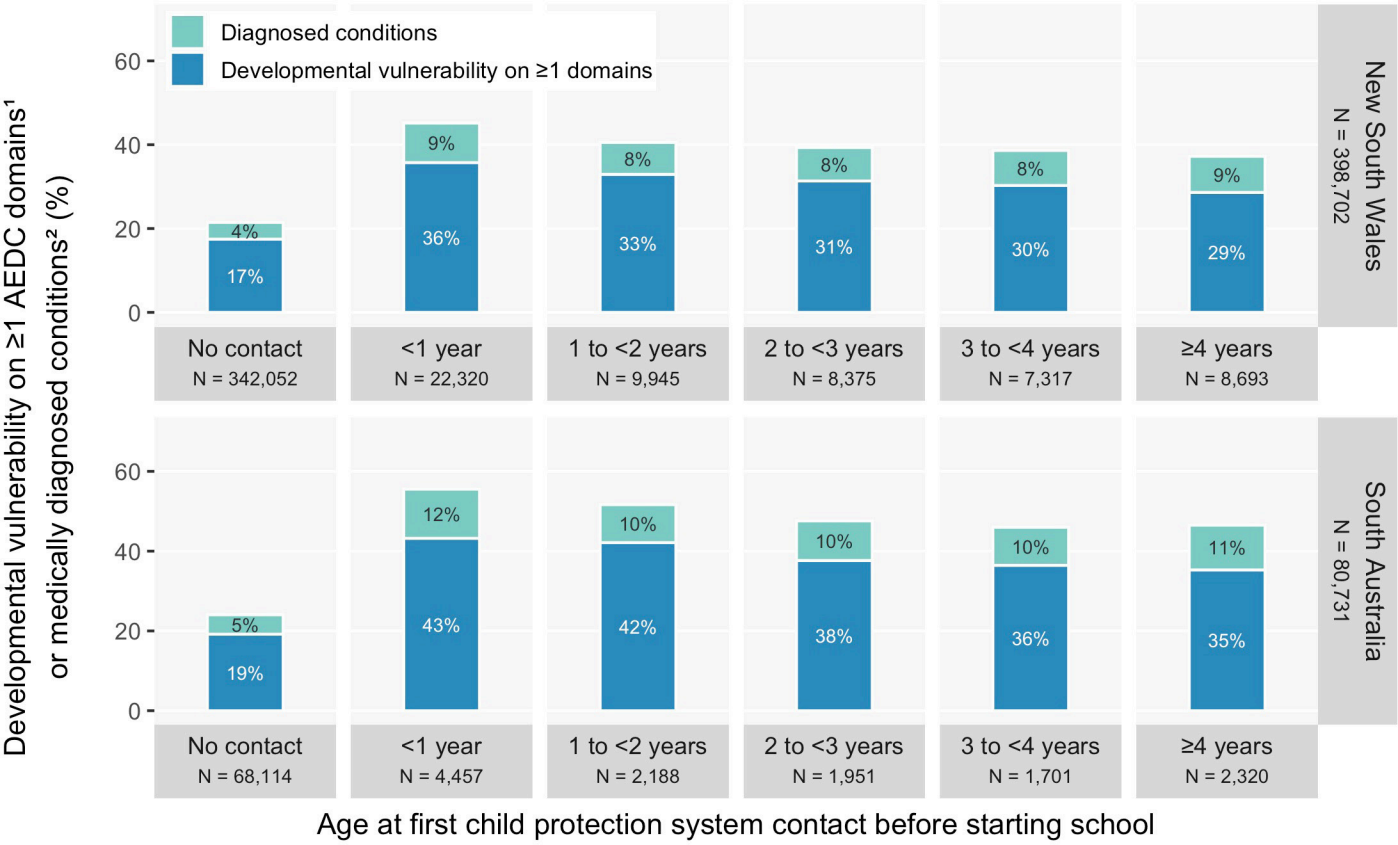
The risk of developmental vulnerability on ≥1 domains^1^ or medically diagnosed conditions^2^ at age 5, by age at first child protection contact before school, among New South Wales and South Australian children in their first year of school (all AEDC years combined^3^) (see data in online supplemental eTable 10). (1) The AEDC DV1 summary indicator was used for the outcome ‘developmental vulnerability on ≥1 AEDC domains’; (2) medically diagnosed conditions with already identified substantial developmental support needs, as listed in online supplemental eTable 2; (3) 2009, 2012, 2015 and 2018 AEDC cycles. AEDC, Australian Early Developmental Census; DV1, developmentally vulnerable on ≥1 AEDC domains.

Figure 2 shows the risk of developmental vulnerability on ≥1 domains was highest among children with their first child protection contact aged <1 year (NSW, 36%; SA, 43%) and 1 to <2 years of age (NSW, 33%; SA, 42%), followed by first contacts aged 2 to <3 years (NSW, 31%; SA, 38%); 3 to <4 years and 4 years. The risk of medically diagnosed conditions was similar across age at first child protection contact groups (NSW, 8–9%; SA, 10–12%), compared with no child protection contact (NSW, 4%; SA, 5%).

#### Number of child protection reports before school

**Figure 3:**
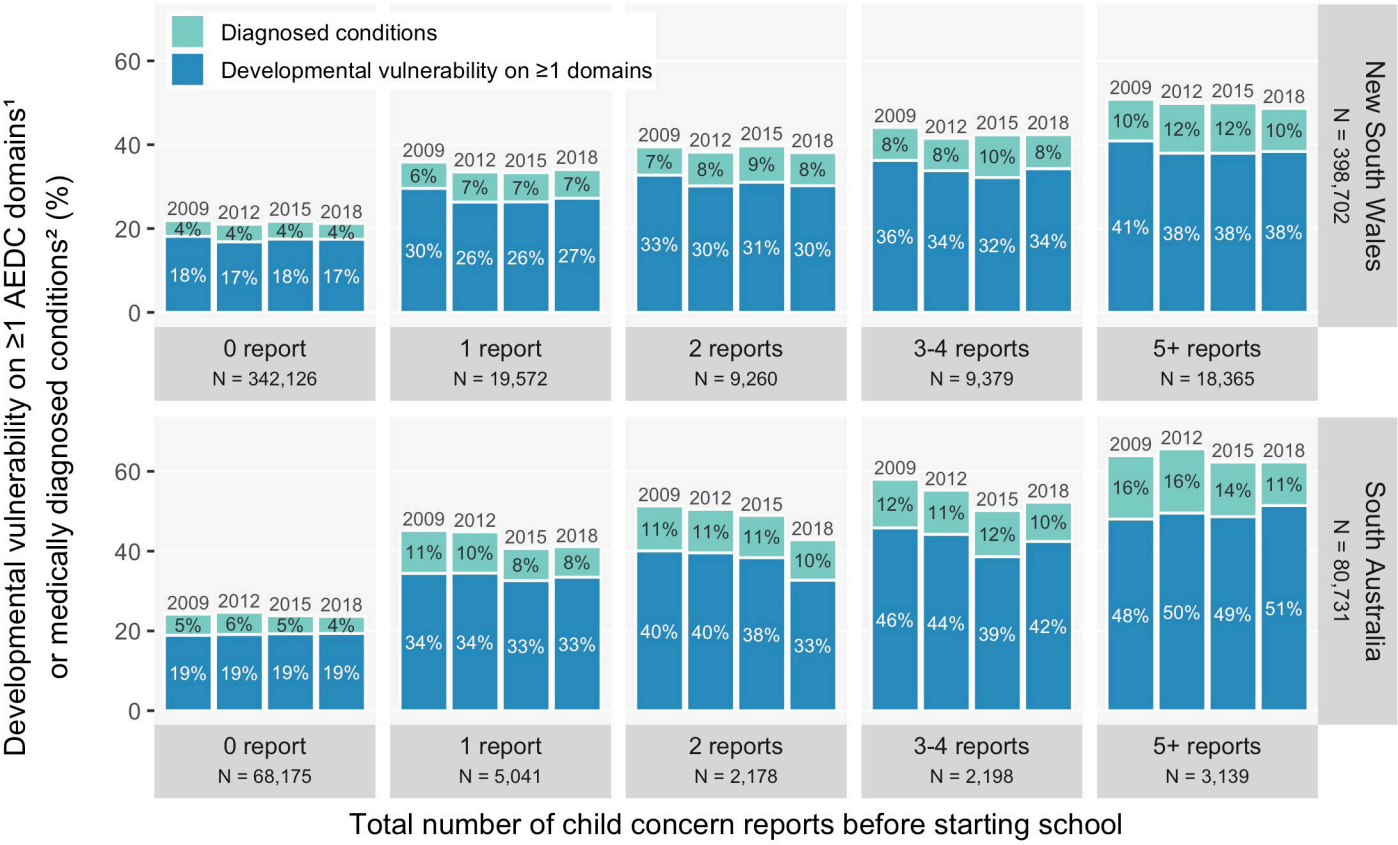
The risk of developmental vulnerability on ≥1 domains^1^ or medically diagnosed conditions^2^ at age 5, by number of child protection reports before school, among New South Wales and South Australian children in their first year of school in 2009, 2012, 2015 or 2018 (see data in online supplemental eTable 11). (1) The AEDC DV1 summary indicator was used for the outcome ‘developmental vulnerability on ≥1 AEDC domains’; (2) medically diagnosed conditions with already identified substantial developmental support needs, as listed in online supplemental eTable 2. AEDC, Australian Early Developmental Census; DV1, developmentally vulnerable on ≥1 AEDC domains.

Figure 3 shows the risk of developmental vulnerability on ≥1 domains increased from 1 report (NSW, 26–30%; SA, 33–34%) through to ≥5 reports (NSW, 38–41%; SA, 48–51%), compared with no child protection contact (NSW, 17–18%; SA, 19%) (online supplemental eFigure 4, eTable 6). Similarly, the risk of medically diagnosed conditions was lowest in those with no child protection contact (NSW, 4%; SA, 4–6%) and increased from 1 report (NSW, 6–7%; SA, 8–11%) to ≥5 reports (NSW, 10–12%; SA, 11–16%).

#### Most serious child protection contact and number of child protection reports before school

**Figure 4:**
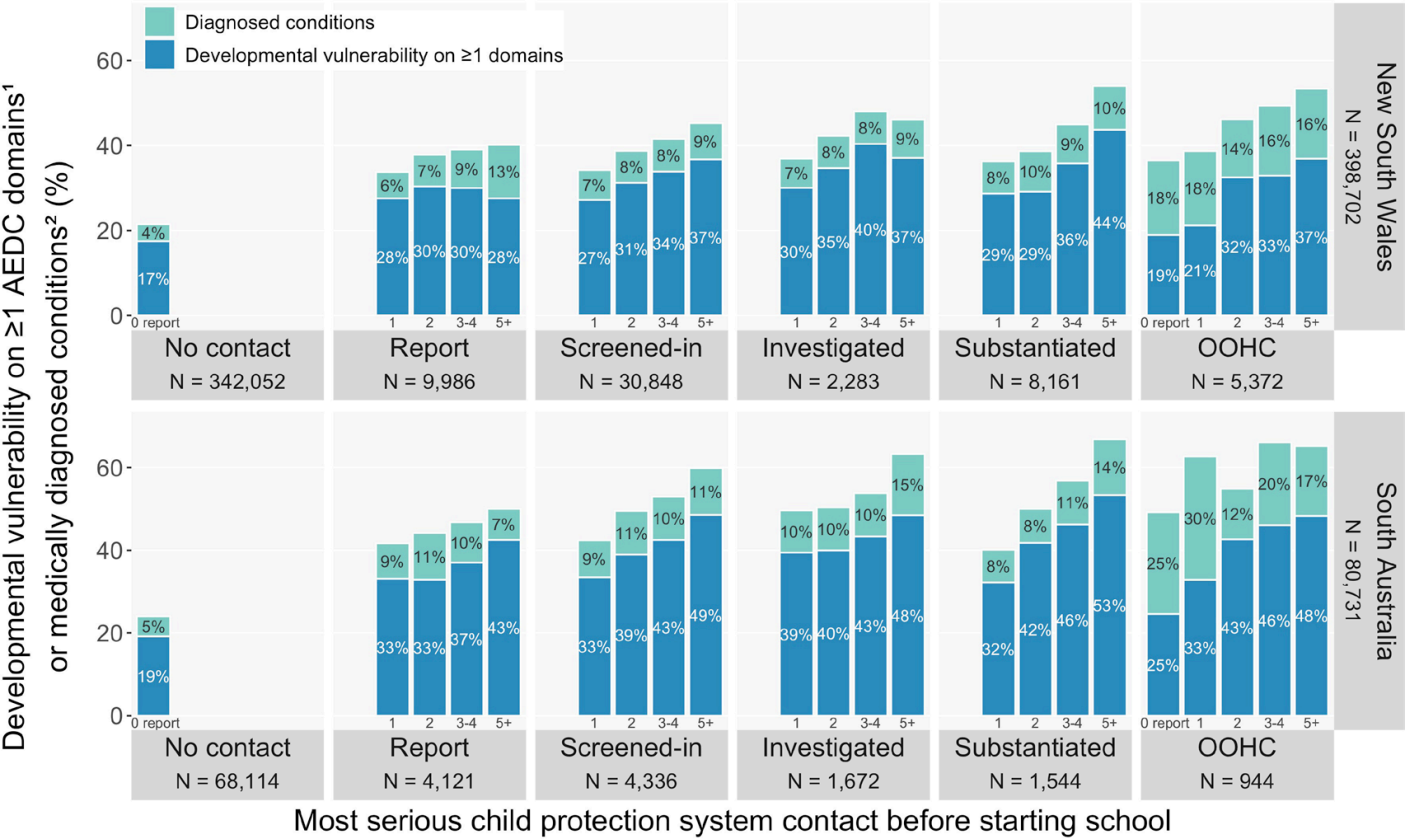
The risk of developmental vulnerability on ≥1 domains^1^ or medically diagnosed conditions^2^ at age 5, by most serious child protection contact and number of child protection reports before school, among New South Wales and South Australian children in their first year of school (all AEDC years combined^3^) (see data in online supplemental eTable 12). Report, child protection report; screened-in, child protection reports that were screened by the system to meet a threshold of risk/harm in each jurisdiction; investigated, child protection services investigation of alleged maltreatment; substantiated, maltreatment that was confirmed by child protection services. The OOHC data includes placements related to care and protection orders, as well as voluntary care, emergency care and respite placements for a short period. (1) The AEDC DV1 summary indicator was used for the outcome ‘developmental vulnerability on ≥1 AEDC domains’; (2) medically diagnosed conditions with already identified substantial developmental support needs, as listed in online supplemental eTable 2; (3) 2009, 2012, 2015 and 2018 AEDC cycles. AEDC, Australian Early Developmental Census; DV1, developmentally vulnerable on ≥1 AEDC domains; OOHC, out-of-home care placement.

Figure 4 shows that, in both jurisdictions, there was a progressively higher risk of developmental vulnerability on ≥1 domains or medically diagnosed conditions among children with more child protection reports (0 to ≥5 reports before school), within each most serious child protection contact group. For example, among children whose most serious contact was a child protection report, the combined developmental risk for both outcomes was 34% (NSW) and 42% (SA) for 1 report only, 38% (NSW) and 44% (SA) for 2 reports and 40% (NSW) and 50% (SA) for ≥5 reports before school. Even children with only one child protection report before school had about 70% higher relative developmental risk than the no child protection group (NSW, 34% vs 21%; SA, 42% vs 24%). A similar pattern of higher developmental risk among children with more reports was observed within each age at first child protection contact group (online supplemental eFigure 5, eTable 7).

### Number and type of developmental domain vulnerabilities, by most serious child protection contact before school

**Figure 5:**
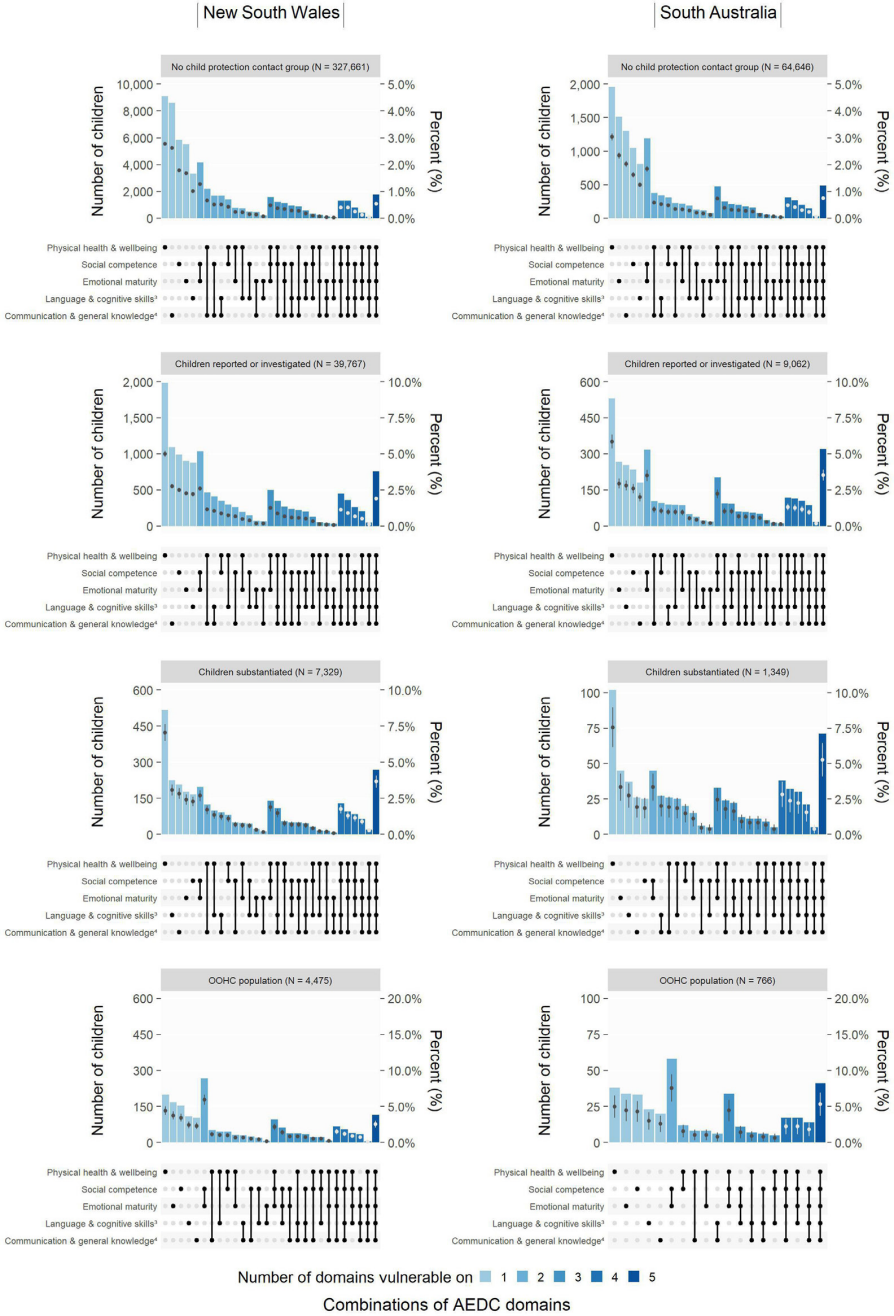
The number (vertical bars) and per cent (circles) of children with the most common combinations of number and type of developmental domain vulnerabilities among New South Wales and South Australian children with valid AEDC domain outcomes^1^ (all AEDC years combined^2^), according to their most serious level of child protection contact before school. The per cent scale (right-hand Y-axis) is comparable across the jurisdictions (see data in online supplemental eTable 13). Reported or investigated, includes all children with a child protection report that was or was not screened-in by the system to meet a threshold of risk/harm in each jurisdiction and/or investigated by child protection services; substantiated, maltreatment that was confirmed by child protection services. The OOHC data includes placements related to care and protection orders, as well as voluntary care, emergency care and respite placements for a short period. (1) Excludes children with previous medically diagnosed conditions because they were not included in the AEDC summary indicators; (2) 2009, 2012, 2015 and 2018 AEDC cycles; (3) full AEDC domain name is ‘Language and cognitive skills (school-based)’; (4) full AEDC domain name is ‘Communication skills and general knowledge’. AEDC, Australian Early Developmental Census; OOHC, out-of-home care placement.

Figure 5 shows similar scales and patterns of the most common combinations of number and type of developmental domain vulnerabilities, according to most serious child protection contact, among 379 232 NSW children and 75 823 SA children with non-missing data for all five AEDC domains (online supplemental eFigures 2-3; analysis sample 2). In both states, developmental vulnerability in the physical health and well-being domain was the most common among children whose most serious child protection contact was: no contact (NSW, 2.8%; SA, 3.0%); child protection report, screened-in and/or investigated (NSW, 5.0%; SA, 5.9%); a substantiation (NSW, 7.1%; SA, 7.6%) (figure 5). Developmental vulnerability in the social competence and emotional maturity domains was the most common combination among the OOHC group in both NSW (5.9%) and SA (7.6%), and one of the most common combinations among other groups. The risk of developmental vulnerability on all five AEDC domains was the second or third most common combination among children with a substantiation or OOHC placement before school.

## Discussion

A key finding from this two-jurisdiction study is that even a single child protection report in the first 2000 days of children’s lives was a robust indicator of developmental risk at age 5. It is perhaps not surprising that children with higher levels of child protection contact, such as substantiations or OOHC, have higher developmental risks, but we also showed that children with only a single report before age 5 without any more serious child protection contact had 1.6–1.8 times higher relative risk of developmental vulnerability on ≥1 domains or medically diagnosed conditions. While these reports to child protection were not considered ‘child protection matters’, they nevertheless carry information on the risk of poor developmental outcomes by age 5. While this may, in part, reflect the higher proportion of socioeconomic and health disadvantage among children in contact with child protection, it nevertheless represents the true population burden. An accurate description of the population burden linked to child protection contact requires crude risk estimates.^16 27^ So beyond its role in ensuring the immediate safety of children, information reported to child protection is also an important source of public health insight that could be better used to inform supportive targeted prevention responses.

Despite different child protection systems and procedures, we found that the scale and patterns of developmental risk among child protection populations were strikingly similar in two Australian jurisdictions and over almost a decade. In both states, one in seven children had a child protection report or more serious contact before school. There was progressively higher developmental risk among whole populations of children with more serious child protection contact, an earlier age at first child protection contact and more child protection reports before starting school. A previous Canadian study also found that children in OOHC had the highest risk of teacher-reported developmental vulnerability; however, children with previous medically diagnosed conditions were excluded from the study population and outcomes were not examined across other levels of child protection system contact.^2^ Even within each of the most serious child protection contact groups in our study, children with more reports had a higher developmental risk. This highlights the strength of the developmental risk signal that child protection reports carry at the population level, beyond the system’s own triaging processes rightly focused on immediate safety.

For the first time, we show that children with medically diagnosed conditions are disproportionately represented in early childhood child protection populations, in addition to children identified as developmentally vulnerable by teachers. Although children with previous medically diagnosed conditions have been assessed by the health system prior to school entry, it is important that their substantial health and development support needs are counted, alongside those with previously unidentified developmental vulnerability, in any data and intelligence that may be used to plan the supportive interventions from the health, child protection, early education and school systems.

This study highlights the importance of ensuring the developmental support needs of children reported to child protection, including First Nations children, are a focus in holistic and culturally responsive prevention approaches to promote the best outcomes for children before starting school. Similar to countries such as Canada, USA and New Zealand,^28^,^31^ First Nations children were over-represented in child protection systems, before starting school, in both Australian states. Systemic racism is a driver of the ‘causes’ of child protection contact, such as poverty, family violence and substance use, and past and continuing child welfare policies and practice resulting in the over-surveillance, reporting and potential pathologising of First Nations children and removal of children from their families.^32 33^ Recent reviews of child protection systems^4 5 34^ and broader policies^35^,^37^ emphasise the need for urgent reform, both jurisdictionally and nationally.

A limitation of this study is that administrative data does not capture all maltreatment experiences or risks experienced by children.^38^ However, it is also the case that adult recall of child maltreatment in interviews and surveys under-ascertains safety concerns reported to child protection.^38^ Given it is not feasible or ethical to collect self-reported maltreatment risk/experience from very young children, child protection administrative data offers the earliest opportunity to inform prevention in whole child populations.

Child protection reports represent an underused asset to plan and deliver—at the earliest possible opportunity—universal and targeted support services for children at higher developmental risk during early childhood. The health system has near-universal reach during pregnancy, birth and the early postnatal period, when it is common to screen for at-risk infants and families and make referrals for assessment and support. Child protection services do not have a statutory responsibility to respond to the unmet needs of children who are not screened in at risk of ‘serious harm’ (safety) or further investigated. However, there is potential to re-envisage the use of child protection reports to inform responses from adjacent agencies, such as the health system, as part of a ‘child health and well-being system’ response to support child development. While progress towards multiagency, whole-population data assets varies across jurisdictions internationally,^39 40^ this study demonstrates the potential to generate timely, contemporary, actionable targets for public health prevention in whole child populations using data on system contacts and outcomes that are routinely collected by different government agencies.

## Conclusion

This two-jurisdiction study has attempted to reframe how data on child protection contacts—experienced by one in seven children by school age—could be used to inform supportive services led by agencies in health, human services and early education and care for children. While the child protection system’s primary function is rightly to deliver statutory responses to safety risks and actual harm, health, human services and early education and care are well placed to deliver non-stigmatising, supportive interventions to children and families with early indicators of developmental risks.

## Supplementary material

10.1136/jech-2024-223006 online supplemental file 1

## Data Availability

Data may be obtained from a third party and are not publicly available.
