# Supplementary material for: Re-envisaging child protection contacts as an early prevention opportunity to support child development and well-being: an Australian data linkage study
Source: J Epidemiol Community Health. 2025 Feb 26;79(7):e223006. doi: 10.1136/jech-2024-223006 (PMC12322469; doi:10.1136/jech-2024-223006)
Supplement: online supplemental file 1 [file jech-79-7-s001.pdf]

Supplementary material

Title: Re-envisaging child protection contacts as an early prevention opportunity to support child development and well-being: an Australian data linkage study.

Contributors: Kathleen Falster[#], Rhiannon Pilkington[#], Tasnia Ahmed*, Alicia Montgomerie*, Mark Hanly, BJ Newton, Marni Brownell, Ben Edwards, Raghu Lingam, Anthony Shakeshaft, Michelle Cretikos, Jessica Stewart Katherine Hawkins, Kitty McLean, John W. Lynch.

#KF and RP contributed equally as first author.
*TA and AM contributed equally to data and analysis.

List of eFigures

eFigure1 Map of Australia with state and territory jurisdictions, including New South Wales and South Australia.. 4
eFigure2 Flow chart representing assembly of study population from linked data sources for children in New South Wales (NSW) Child E-Cohort Project platform. 6
eFigure3 Flow chart representing assembly of study population from linked source data for children in the South Australian (SA) BEBOLD platform.. 7
eFigure4 The risk of developmental vulnerability on ≥1 domains[1] or medically diagnosed conditions2 at school entry, by number of child protection reports before school, among New South Wales and South Australian children in their first year of full-time school (all AEDC years combined[1]). 22
eFigure5 The risk of developmental vulnerability on ≥1 domain[1] or medically diagnosed conditions[2] at school entry, by age at first child protection contact and number of child protection reports before school, among New South Wales and South Australian children in their first year of full-time school (all AEDC years combined[1]). 24

List of eTables

eTable1 Jurisdiction-specific and harmonised terms and definitions for child protection contacts recorded in administrative datasets in New South Wales and South Australia. 5
eTable2 Medically diagnosed conditions with special needs[1] information for the 2009-2018 AEDC cycles. 8
eTable3 AEDC year by the most serious type of child protection contact before school entry in New South Wales and South Australia. 9
eTable4a. Child protection reports and screened-in reports recorded during and after incomplete data period (2009 AEDC cohort), or equivalent periods (2012-2018 AEDC cohorts) in New South Wales. 14
eTable5 Demographic characteristics by most serious type of child protection contact before school entry, in New South Wales and South Australia, all AEDC years combined 17
eTable6 The risk of developmental vulnerability on ≥1 domain[1] or medically diagnosed conditions[2] at school entry, by number of child protection reports before school, among New South Wales and South Australia children in their first year of full-time school (all AEDC years combined[1]) (data table for eFigure4). 23
eTable7 The risk of developmental vulnerability on ≥1 domain[1] or medically diagnosed conditions[2] at age five, by age at first child protection contact and number of child protection reports before school, among New South Wales and South Australian children in their first year of school (all AEDC years combined[3]) (data table for eFigure5). 25
eTable8 The risk of developmental vulnerability on ≥1 AEDC domain[1] or medically diagnosed conditions[2], by most serious child protection contact before school, among New South Wales and South Australian children in their first year of full-time school in 2009, 2012, 2015 or 2018. (data table for Figure 1a). 27
eTable9 The risk of developmental vulnerability on 1-5 AEDC domains, or medically diagnosed conditions[1], by most serious child protection contact before school, among New South Wales and South Australian children in their first year of full-time school in 2009, 2012, 2015 or 2018. (data table for Figure 1b). 29
eTable10 The risk of developmental vulnerability on ≥1 domain[1] or medically diagnosed conditions[2] at age five, by age at first child protection contact before school, among New South Wales and South Australian children in their first year of school (all AEDC years combined[3]) (data table for Figure 2). 31
eTable11 The risk of developmental vulnerability on ≥1 domain[1] or medically diagnosed conditions[2] at age five, by number of child protection reports before school, among New South Wales and South Australian children in their first year of school in 2009, 2012, 2015 or 2018 (data table for Figure 3). 32
eTable12 The risk of developmental vulnerability on ≥1 domain[1] or medically diagnosed conditions[2] at age five, by most serious child protection contact and number of child protection reports before school, among New South Wales and South Australian children in their first year of school (all AEDC years combined[3]). (data table for Figure 4). 34
eTable13 The number and percent of children with the most common combinations of number and type of developmentally vulnerable domains among New South Wales and South Australian children with valid AEDC domain outcomes[1] (combined for all AEDC years[2]), according to their most serious level of child protection contact by their first year of full-time school. The percent scale (right-hand Y-axis) is comparable across the jurisdictions. (data table for Figure 5). 36

SUPPLEMENTARY MATERIAL

eFigure1 Map of Australia with state and territory jurisdictions, including New South Wales and South Australia.

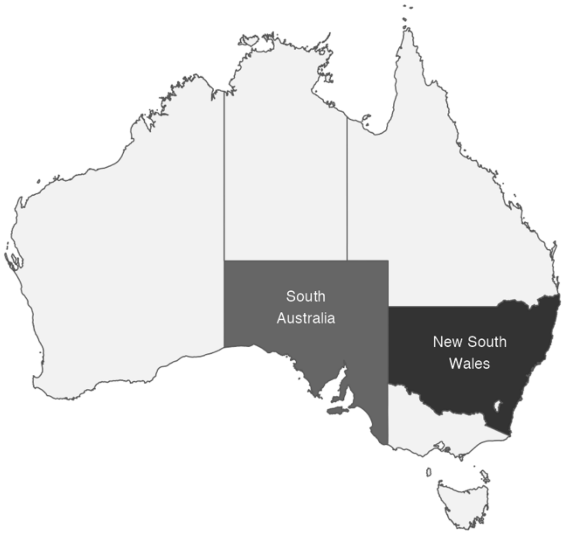

eTable1 Jurisdiction-specific and harmonised terms and definitions for child protection contacts recorded in administrative datasets in New South Wales and South Australia.

New South Wales	South Australia[4]
**Child protection report**	
Child concern report: A report to the Child Protection Helpline from any member of the community, including mandatory reporters, who suspect, on reasonable grounds, that a child or young person is at risk of significant harm.[1]	*Notification:* A report to the Child Protection Agency concerning suspected child abuse or neglect.
**Screened-in child protection report**	
Risk of significant harm (ROSH) report: When reports are made to the Child Protection Helpline, a child protection caseworker will assess what action needs to be taking using a screening tool. A child or young person is considered to be at risk of significant harm if the circumstances that are causing concern for their safety, welfare or wellbeing are present to a significant extent. This means it is sufficiently serious to warrant a response by a statutory authority, irrespective of a family's consent. In January 2010, the threshold for screening in reports changed from 'risk of harm' to 'risk of significant harm'.[1,2]	*Screened-in notification:* A term used to indicate whether the situation described by the caller (notifier) is of sufficient concern to warrant intervention by the Child Protection Agency. Those that meet the threshold are screened in; those that do not are screened out.
**Investigation**	
Field assessment: An investigation and assessment to determine whether the child or young person is at risk of significant harm, conducted by a child protection caseworker.	*Investigation:* A determination of whether an incident of child abuse or neglect has occurred, and the circumstances of its occurrence.
**Substantiation**	
Substantiation: Actual harm or risk of harm was substantiated from the field assessments.	*Substantiation:* At the end of an investigation, the practitioner (in consultation with the supervisor) decides whether abuse or neglect has occurred or risk of abuse or neglect and records this decision, which is known as 'substantiation'. Substantiation is a professional judgment that must be supported by sound rationale based on the information gathered.
**Out-of-home care (OOHC) placement**	
OOHC placement: Out-of-home care means residential care and control of a child or young person that is provided—(a) by a person other than a parent of the child or young person, and (b) at a place other than the usual home of the child or young person, whether or not for fee, gain or reward.[2] This can include care with a relative, a friend, a foster carer, residential care or independent living arrangements.	*OOHC placement:* System of caring for a child who is removed from their family of origin. Includes (but is not limited to) home-based care, emergency care and residential care.

1. NSW Government Department of Communities and Justice Deciding to make a report to the Child Protection Helpline webpage (https://www.facs.nsw.gov.au/families/Protecting-kids/reporting-child-at-risk/should-i-call); 2. NSW Government Children and Young Persons (Care and Protection) Act 1998 No 157 (https://legislation.nsw.gov.au/view/html/inforce/current/act-1998-157); 3. NSW Government Department of Communities and Justice What Happens once a report is made to the Child Protection Helpline? Webpage (https://www.facs.nsw.gov.au/families/Protecting-kids/reporting-child-at-risk/what-next); 4. Source of definitions is the Glossary section of the Child Protection Systems Royal Commission, The life they deserve: Child Protection Systems Royal Commission Report, Government of South Australia, 2016.(https://www.childprotection.sa.gov.au/documents/report/child-protection-systems-royal-commission-report.pdf).

SUPPLEMENTARY MATERIAL

eFigure2 Flow chart representing assembly of study population from linked data sources for children in New South Wales (NSW) Child E-Cohort Project platform.

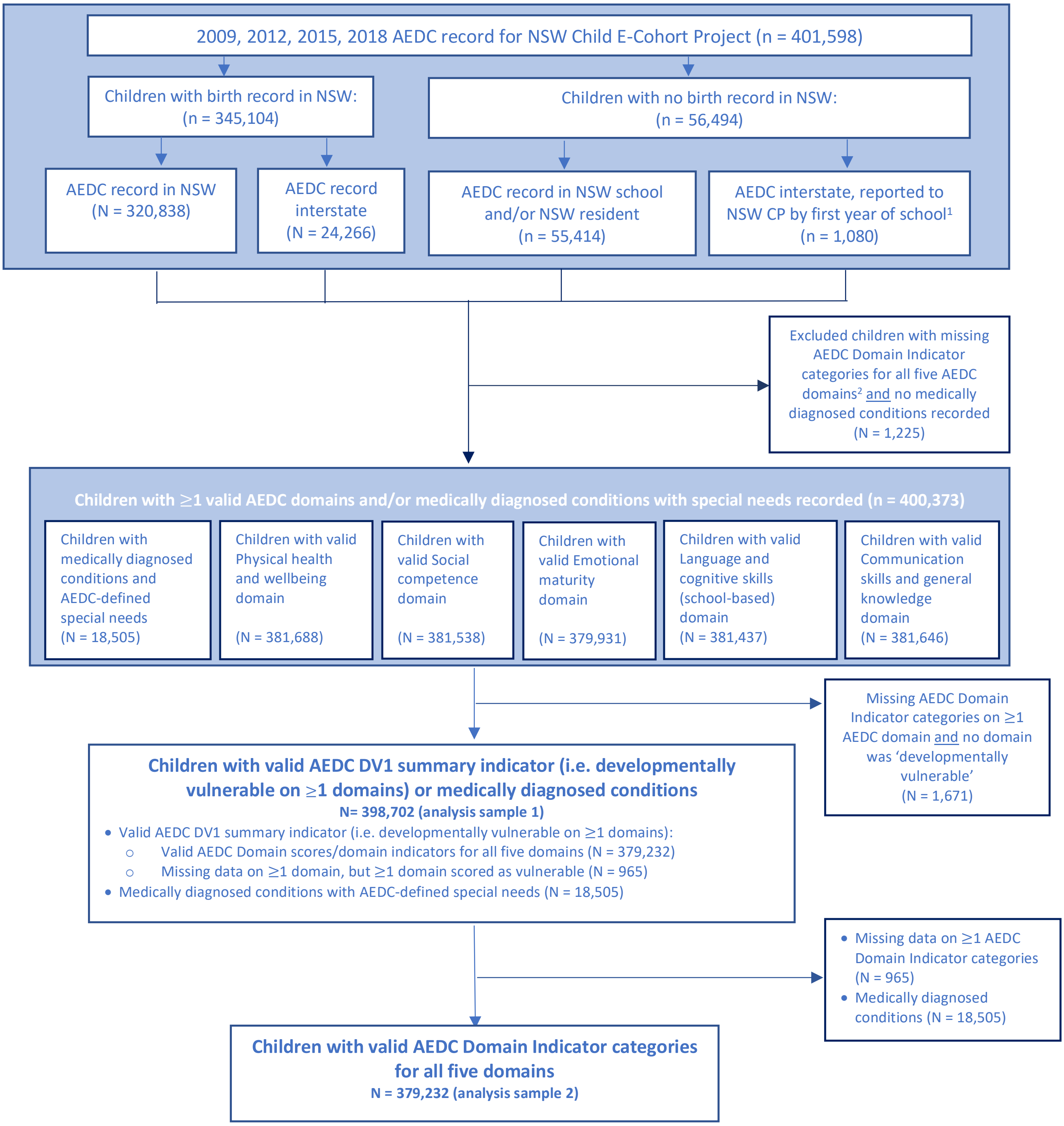

AEDC, Australian Early Development Census; CP, child protection; NSW, New South Wales. 1. Child protection records on or prior to 31 January in first year of full-time school. 2. AEDC Domain indicator categories (including Developmentally vulnerable) are not calculated for children with missing (or invalid) domain scores.

SUPPLEMENTARY MATERIAL

eFigure3 Flow chart representing assembly of study population from linked source data for children in the South Australian (SA) BEBOLD platform

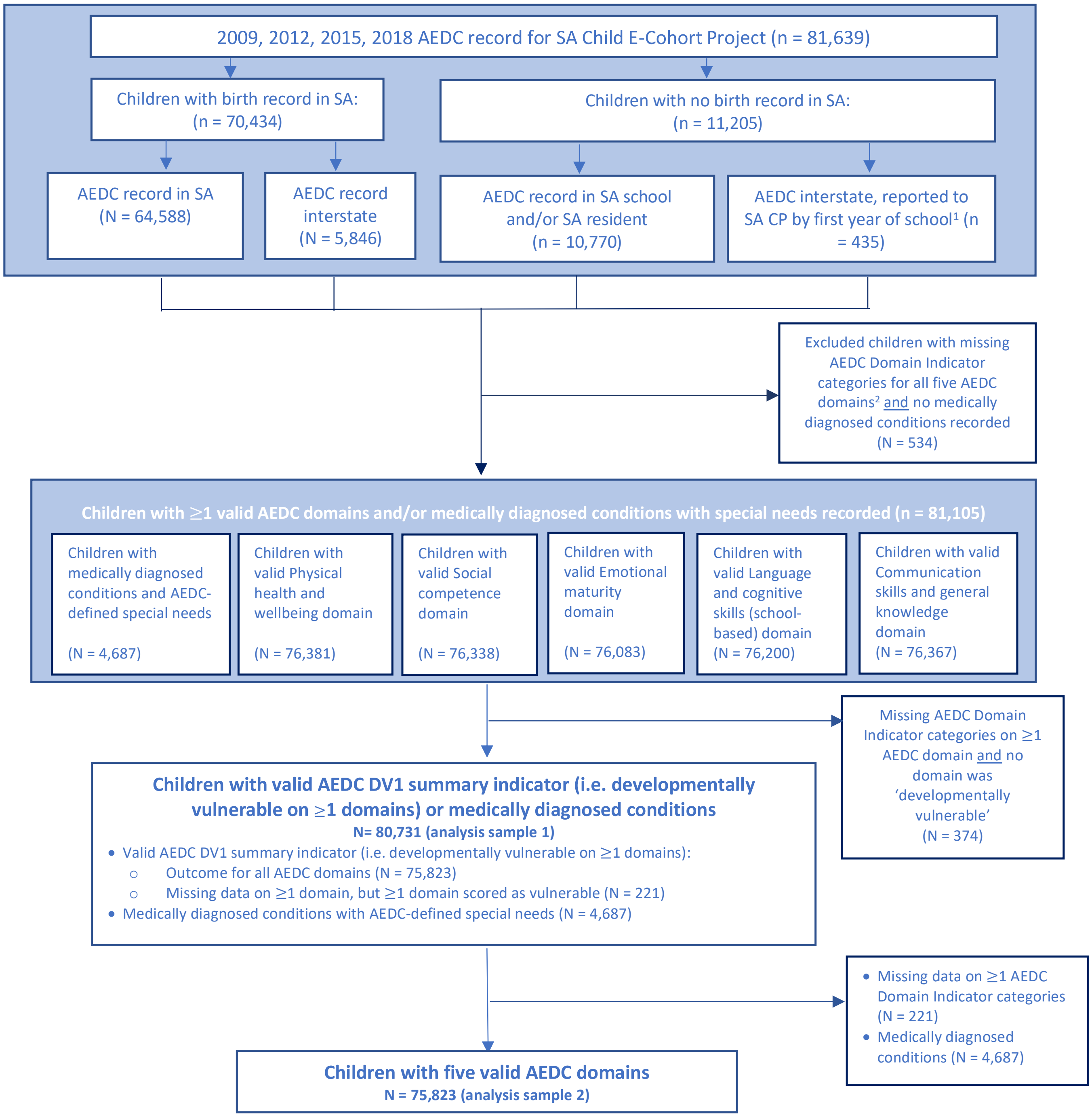

AEDC, Australian Early Development Census; BEBOLD, Better Evidence, Better Outcomes, Linked Data; CP, child protection; NSW, New South Wales. 1. Child protection records on or prior to 31 January in first year of full-time school. 2. AEDC Domain indicator categories (including Developmentally vulnerable) are not calculated for children with missing (or invalid) domain scores.

eTable2 Medically diagnosed conditions with special needs[1] information for the 2009-2018 AEDC cycles.

2009-2012 AEDC cycles: Children were classified as having medically diagnosed 'special needs' if it was reported that the child required special assistance because of chronic medical, physical, or intellectually disabling conditions (e.g. Autism, Cerebral palsy, Down syndrome), based on a medical diagnosis. No specific diagnosis information was recorded in the AEDC data collections in the 2009 and 2012 cycles.

2015-2018 AEDC cycles: Children were classified as having 'medically diagnosed special needs' if they had a medically diagnosed condition/s recorded, plus 'special needs' indicated. eTable2 summarises the most common medically diagnosed conditions recorded in the 2015 and 2018 AEDC cycles for children in NSW[2] and SA.

	New South Wales						South Australia					
	2015 AEDC		2018 AEDC		2015 & 2018 AEDC		2015 AEDC		2018 AEDC		2015 & 2018 AEDC	
	n	%	n	%	n	%	n	%	n	%	n	%
**Number and percent of children with one, two, or three or more medically diagnosed conditions recorded in the AEDC**												
One	3,966	79.9	3,425	75.7	7,391	77.9	954	82.2	880	79.3	1,834	80.8
Two	813	16.4	843	18.6	1,656	17.5	160	13.8	175	15.8	335	14.8
Three or more	182	3.7	254	5.6	436	4.6	46	4.0	55	5.0	101	4.4
**Number and percent of children with the most prevalent medically diagnosed conditions recorded in the AEDC[3]**												
Autism spectrum disorders (ASD)[4]	1,864	37.6	2,231	49.3	4,095	43.2	428	36.9	571	51.4	999	44.0
Attention Deficit Hyperactivity Disorder (ADHD)	469	9.5	586	13.0	1,055	11.1	53	4.6	52	4.7	105	4.6
Global Developmental Delay	504	10.2	378	8.4	882	9.3	227	19.6	207	18.6	434	19.1
Asthma	486	9.8	335	7.4	821	8.7	76	6.6	69	6.2	145	6.4
Anaphylaxis	285	5.7	211	4.7	496	5.2	32	2.8	27	2.4	27	1.2
Cerebral Palsy	167	3.4	144	3.2	311	3.3	48	4.1	23	2.1	71	3.1
Epilepsy	142	2.9	131	2.9	273	2.9	31	2.7	22	2.0	53	2.3
Deaf	145	2.9	124	2.7	269	2.8	38	3.3	31	2.8	69	3.0
Anxiety	59	1.2	123	2.7	182	1.9	5	0.4	26	2.3	31	1.4
Dyspraxia	110	2.2	69	1.5	179	1.9	16	1.4	12	1.1	28	1.2
Down Syndrome	68	1.4	66	1.5	134	1.4	14	1.2	13	1.2	27	1.2
Diabetes	62	1.2	63	1.4	125	1.3	7	0.6	16	1.4	23	1.0
Blind	35	0.7	33	0.7	68	0.7	7	0.6	16	1.4	23	1.0
Foetal Alcohol Spectrum Disorder (FASD)	24	0.5	24	0.5	48	0.5	7	0.6	7	0.6	14	0.6
Other[5]	1,915	38.6	1,649	36.5	3,564	37.6	386	33.3	293	26.4	679	29.9
**Total children**	**4,961**	**100**	**4,522**	**100**	**9,483**	**100**	**1,160**	**100**	**1,110**	**100**	**2,270**	**100**

AEDC, Australian Early Development Census. 1. In this study, we used the term medically diagnosed conditions with higher support needs because many affected children, families and communities prefer not to use the term 'special needs'; 2. Based on the medically diagnosed condition variables in the 2015 and 2018 AEDC linked data, released for the NSW Child E-Cohort Project in 2024; 3. Children may have more than one medically diagnosed condition recorded; the groups are not mutually exclusive; 4. Autism Spectrum Disorders (ASD) includes the following other terms recorded in the data: Autism, ASD and Asperger syndrome; 5. Other includes medically diagnosed conditions recorded as 'Other' (i.e. not specified), as well as conditions with <5 children, including: Selective Mutism, Eczema / Dermatitis, Muscular Dystrophy, Cleft Palate, Cystic Fibrosis, Fragile X, Coeliac / Celiac, Leukemia, Oppositional Defiance Disorder/Oppositional Defiant Disorder/ODD, Spina bifida, Hydrocephalus, Neurofibromatosis, Otitis Media, Perthes Disease and Dyslexia. In the 2015 and 2018 AEDC cycles combined, the 3,564 other conditions were recorded for 3,106 children in NSW, while the 679 other conditions were recorded for 676 children in SA.

eTable3 AEDC year by the most serious type of child protection contact before school entry in New South Wales and South Australia.

	New South Wales							South Australia						
	Most serious type of child protection contact before school age							Most serious type of child protection contact before school age						
	No contact n (Row %)	Reported n (Row %)	Screened-in n (Row %)	Investigated n (Row %)	Substantiated n (Row %)	OOHC n (Row %)	Total N (Row %)	No contact n (Row %)	Reported n (Row %)	Screened-in n (Row %)	Investigated n (Row %)	Substantiated n (Row %)	OOHC n (Row %)	Total N (Row %)
**AEDC Year**														
2009	79,173 (84.6)	2,033 (2.2)	9,378 (10.0)	270 (0.3)	1,581 (1.7)	1,176 (1.3)	93,611 (100.0)	15,713 (87.0)	602 (3.3)	755 (4.2)	442 (2.4)	326 (1.8)	216 (1.2)	18,054 (100.0)
2012	83,855 (84.0)	2,427 (2.4)	9,770 (9.8)	269 (0.3)	1,993 (2.0)	1,464 (1.5)	99,778 (100.0)	16,873 (84.6)	856 (4.3)	1109 (5.6)	491 (2.5)	398 (2.0)	226 (1.1)	19,953 (100)
2015	88,116 (86.9)	2,807 (2.8)	6,407 (6.3)	519 (0.5)	2,191 (2.2)	1,334 (1.3)	101,374 (100.0)	17,555 (83.4)	1,159 (5.5)	1222 (5.8)	436 (2.1)	444 (2.1)	230 (1.1)	21,046 (100)
2018	90,908 (87.5)	2,719 (2.6)	5,293 (5.1)	1,225 (1.2)	2,396 (2.3)	1,398 (1.4)	103,939 (100.0)	17,973 (82.9)	1,504 (6.9)	1250 (5.8)	303 (1.4)	376 (1.7)	272 (1.3)	21,678 (100)
**Total**	**342,052 (85.8)**	**9,986 (2.5)**	**30,848 (7.7)**	**2,283 (0.6)**	**8,161 (2.0)**	**5,372 (1.4)**	**398,702 (100.0)**	**68,114 (84.4)**	**4,121 (5.1)**	**4,336 (5.4)**	**1,672 (2.1)**	**1,544 (1.9)**	**944 (1.2)**	**80,731 (100.0)**

AEDC, Australian Early Development Census; OOHC, Out-of-home care.

Supplement 4. Sensitivity analysis to estimate scale of under-ascertainment of child protection reports and investigations among NSW children who started school in 2009.

Background and rationale for sensitivity analysis:
In New South Wales (NSW), statewide child protection report data were recorded in the Key Information Directory System (KiDS) from November 1 2003, and investigation data were incomplete prior to January 1 2005. In the 2009 NSW AEDC cohort, about two-thirds were born before child protection reports were recorded in the KiDS and 99% were born in the period of incomplete investigation data. For this reason, there is likely under-ascertainment of child protection reports and investigations in the 2009 NSW AEDC cohort for the prenatal period until about two years of age. Although this under-ascertainment may have resulted in misclassification of early life child protection contacts, some of the children may have been re-reported or re-investigated before they started school. To understand the potential degree of misclassification of child protection contacts before starting school in the 2009 NSW AEDC cohort, we examined first reports/investigations in periods equivalent to the incomplete data period for the 2009 AEDC cohort and re-reports/investigations before school in the 2012-2018 AEDC cohorts in NSW.

Methods:

2009 NSW AEDC cohort (impacted by incomplete data)
Of the 93,611 children in the 2009 NSW AEDC cohort, 67% (N=62,505) were born in the incomplete data period for child protection reports (i.e. before January 1 2004) and 99% (N=93,344) were born in the incomplete data period for investigations (i.e. before January 1 2005). Among the 2009 AEDC cohort children born in the incomplete data periods, we calculated the number and percent of children with ≥1 child protection reports, screened-in reports, investigations, and substantiations (outcome of investigations) before starting school.

2012, 2015, 2018 NSW AEDC cohorts (not impacted by incomplete data)
Using data from the 2012, 2015 and 2018 NSW AEDC cohorts, we defined a group of children who were born in the period equivalent to the incomplete data periods for reports and investigations for the 2009 AEDC cohort. In this group, we calculated the number and percent of children with child protection reports, screened-in reports, investigations and substantiations: (i) before starting school (to compare to above); (ii) first-recorded in the incomplete data period equivalent (to estimate the degree of early life under-ascertainment in the 2009 AEDC cohort); and (iii) re-recorded and not re-recorded after the incomplete data period equivalent (to estimate the degree of later ascertainment of child protection contacts by school age). The <u>not</u> re-recorded group represent an estimate of the number of children with missing child protection contacts in the 2009 AEDC cohort.

Estimated under-ascertainment of child protection contacts in the 2009 AEDC cohort by school entry
To estimate the scale of under-ascertainment of child protection contacts in the 2009 AEDC cohort due to incomplete data, we: (i) added the numbers of children <u>not</u> re-reported or re-investigated to child protection before starting school in the 2012-2018 AEDC cohorts to the total number of children with the same child protection contacts in the 2009 AEDC cohort (referred to as the ‘adjusted 2009’ estimates); (ii) calculated the percent of children in the 2009 AEDC cohort with the ‘adjusted 2009’ estimates for each contact. The ‘adjusted 2009’ percent was then compared to the actual observed percent for each child protection contact.

Results:

Child protection reports

<u>Children born during the incomplete data period (2009) or equivalent (2012-2018)</u>
There were 9,201 (15%) of children with ≥1 child protection reports before starting school among the 62,505 children born during the incomplete data period in the 2009 AEDC cohort, which was similar or higher than the 13% to 16% among the children born during the incomplete data period equivalent in the 2012-2018 AEDC cohorts (Table S4a).

Among the 9,387 to 10,634 children reported before starting school in the 2012, 2015 and 2018 AEDC cohorts, 27% to 38% children were first reported during the incomplete data period equivalent. Of these, 74% to 81% of children were re-reported and 19% to 26% were not re-reported to child protection before starting school.

To estimate the potential scale of under-ascertainment of child protection reports in the children born during the incomplete data period in the 2009 AEDC cohort, we separately added the number of children not re-reported in the 2012-2018 AEDC cohorts (N ranging 552 to 946) to the 9,201 children in the 2009 AEDC cohort with ≥1 child protection reports before starting school. Using this 'adjusted 2009' number of children with ≥1 child protection reports before school, we estimated that 16% (9,796 to 10,147/62,505) of children born during the incomplete data period in the 2009 AEDC cohort may have had ≥1 child protection reports before starting school. This suggests there was 1 percentage point under-ascertainment of the percent of children with ≥1 child protection reports among those born during the incomplete data period in the 2009 AEDC cohort due to incomplete child protection report data in early life (i.e. 16% versus 15%).

Children born after the incomplete data period (2009) or equivalent (2012-2018)
Among the 2009 AEDC cohort, the percent of children with ≥1 child protection reports before starting school was 17% among those born after the incomplete data period, respectively (Table S4a). The percent of children with ≥1 child protection *before starting school* were higher in the 2009 AEDC cohort than the 2012-2018 AEDC cohorts among children born after the incomplete data period equivalent (12 to 16%).

Among the 3,428 to 5,279 children reported before starting school in the 2012, 2015 and 2018 AEDC cohorts, 9% to 13% children were first reported during the incomplete data period equivalent. These reports reflect prenatal reports prior to the child's date of birth, which was after the incomplete data period. Of these, 88% to 93% of children were re-reported and 7% to 12% were not re-reported to child protection before starting school.

To estimate the potential scale of under-ascertainment of child protection reports in the children born after the incomplete data period in the 2009 AEDC cohort, we separately added the number of children not re-reported in the 2012-2018 AEDC cohorts (N ranging 28 to 62) to the 5,194 children in the 2009 AEDC cohort with ≥1 child protection reports before starting school. Using this 'adjusted 2009' number of children with ≥1 child protection reports before school, we estimated that 17% (5,222 to 5,256/31,106) of children born after the incomplete data period in the 2009 AEDC cohort may have had ≥1 child protection reports before starting school. This suggests there was <1 percentage point under-ascertainment of the percent of children with ≥1 child protection reports among those born after the incomplete data period in the 2009 AEDC cohort due to incomplete child protection report data in the prenatal period (i.e. 17% versus 17%).

Screened-in child protection reports

Children born during the incomplete data period (2009) or equivalent (2012-2018)
There were 7,918 (13%) of children with ≥1 screened-in child protection reports before starting school among the 62,505 children born during the incomplete data period in the 2009 AEDC cohort, which was similar to or higher than the 10% to 13% among the children born during the incomplete data period equivalenting the 2012-2018 AEDC cohorts (Table S4a).

Among the 7,492 to 9,008 children reported before starting school in the 2012, 2015 and 2018 AEDC cohorts, 24% to 44% children were first reported during the incomplete data period equivalent. Of these, 65% to 78% of children were re-reported and 22% to 35% were not re-reported to child protection before starting school.

To estimate the potential scale of under-ascertainment of screened-in child protection reports in the children born during the incomplete data period in the 2009 AEDC cohort, we separately added the number of children not re-reported in the 2012-2018 AEDC cohorts (N ranging 509 to 1,155) to the 7,918 children in the 2009 AEDC cohort with ≥1 screened-in child protection reports before starting school. Using this 'adjusted 2009' number of children with ≥1 screened-in child protection reports before school, we estimated that 13% to 15% (8,427 to 9,073/62,505) of children born during the incomplete data period in the 2009 AEDC cohort may have had ≥1 screened-in child protection reports before starting school. This suggests there was <1-2 percentage points under-ascertainment of the percent of children with ≥1 screened-in child protection reports among those born during

the incomplete data period in the 2009 AEDC cohort due to incomplete child protection report data in early life (i.e. 13-15% versus 13%).

Children born after the incomplete data period (2009) or equivalent (2012-2018)
Among the 2009 AEDC cohort, the percent of children with ≥1 screened-in child protection reports before starting school was 14% among those born after the incomplete data period, respectively (Table S4a). The percent of children with ≥1 screened-in child protection before starting school were higher in the 2009 AEDC cohort than the 2012-2018 AEDC cohorts among children born after the incomplete data period equivalent (10% to 14%).

Among the 2,687 to 4,470 children reported before starting school in the 2012, 2015 and 2018 AEDC cohorts, 9% to 16% children were first screened-in during the incomplete data period equivalent. These reports reflect prenatal reports prior to the child's date of birth, which was after the incomplete data period. Of these, 80% to 89% of children were re-screened-in and 11% to 20% were not re-screened-in to child protection before starting school.

To estimate the potential scale of under-ascertainment of screened-in child protection reports in the children born after the incomplete data period in the 2009 AEDC cohort, we separately added the number of children not re-screened-in in the 2012-2018 AEDC cohorts (N ranging 28 to 95) to the 4,420 children in the 2009 AEDC cohort with ≥1 screened-in child protection reports before starting school. Using this 'adjusted 2009' number of children with ≥1 screened-in child protection reports before school, we estimated that 14% to 15% (4,448 to 4,515/31,106) of children born after the incomplete data period in the 2009 AEDC cohort may have had ≥1 screened-in child protection reports before starting school. This suggests there was ≤1 percentage point under-ascertainment of the percent of children with ≥1 screened-in child protection reports among those born after the incomplete data period in the 2009 AEDC cohort due to incomplete child protection report data in the prenatal period (i.e. 14-15% versus 14%).

Investigations

Children born during the incomplete data period (2009) or equivalent (2012-2018)
There were 2,751 (3%) of children with ≥1 investigations before starting school among the 93,011 children born during the incomplete data period in the 2009 AEDC cohort, which was 1-2 percentage points less than the 4% to % among the children born during the incomplete data period equivalenting the 2012-2018 AEDC cohorts (Table S4b).

Among the 3,601 to 4,969 children investigated before starting school in the 2012, 2015 and 2018 AEDC cohorts, 37% to 44% of children were first investigated during the incomplete data period equivalent. Of these, 37% to 44% of children were re-investigated and 56% to 61% were not re-investigated to child protection before starting school.

To estimate the potential scale of under-ascertainment of investigations in the children born during the incomplete data period in the 2009 AEDC cohort, we separately added the number of children not re-investigated in the 2012-2018 AEDC cohorts (N ranging 884 to 1,092) to the 2,751 children in the 2009 AEDC cohort with ≥1 investigations before starting school. Using this 'adjusted 2009' number of children with ≥1 investigations before school, we estimated that 4% (3,635 to 3,843/93,011) of children born during the incomplete data period in the 2009 AEDC cohort may have had ≥1 investigations before starting school. This suggests there was 1 percentage point under-ascertainment of the percent of children with ≥1 investigations among those born during the incomplete data period in the 2009 AEDC cohort due to incomplete child protection report data in early life (i.e. 4% versus 3%).

Children born after the incomplete data period (2009) or equivalent (2012-2018)
Less than 1% of children (N ranging 15-34) were born after the incomplete data period equivalent in the 2012-2018 AEDC cohorts. As such, the numbers were too small to assess first-time and re-investigations.

Substantiations

Children born during the incomplete data period (2009) or equivalent (2012-2018)

There were 2,416 (3%) of children with ≥1 substantiations before starting school among the 93,011 children born during the incomplete data period in the 2009 AEDC cohort, which was similar to or less than the 3% to 4% among the children born during the incomplete data period equivalenting the 2012-2018 AEDC cohorts (Table S4b).

Among the 3,296 to 3,687 children reported before starting school in the 2012, 2015 and 2018 AEDC cohorts, 39% to 46% of children were first reported during the incomplete data period equivalent. Of these, 33% to 36% of children were re-reported and 64% to 67% were not re-reported to child protection before starting school.

To estimate the potential scale of under-ascertainment of substantiations in the children born during the incomplete data period in the 2009 AEDC cohort, we separately added the number of children not re-reported in the 2012-2018 AEDC cohorts (N ranging 865 to 1,136) to the 2,416 children in the 2009 AEDC cohort with ≥1 substantiations before starting school. Using this 'adjusted 2009' number of children with ≥1 substantiations before school, we estimated that 4% (3,281 to 3,552/93,011) of children born during the incomplete data period in the 2009 AEDC cohort may have had ≥1 substantiations before starting school. This suggests there was 1 percentage points under-ascertainment of the percent of children with ≥1 substantiations among those born during the incomplete data period in the 2009 AEDC cohort due to incomplete child protection report data in early life (i.e. 4% versus 3%).

Children born after the incomplete data period (2009) or equivalent (2012-2018)

Less than 1% of children (N ranging 15 to 34) were born after the incomplete data period equivalent in the 2012-2018 AEDC cohorts. As such, the numbers were too small to assess first-time and re-substantiations.

Summary of findings:

Our sensitivity analyses suggest there may have been <1-2 percentage points under-ascertainment of child protection reports and/or screened in child protection reports among those born during and after the incomplete data period in the 2009 AEDC cohort, due to incomplete child protection report data in the prenatal period and/or first year of life. For investigations and substantiations, we estimated there may have been 1 percentage point under-ascertainment among children born during the incomplete data period in the 2009 AEDC cohort (99% of the cohort). The under-ascertainment of some child protection contacts in the 2009 AEDC cohort may have resulted in misclassification of children in the: no child protection contact or less serious child protection contact groups; fewer numbers of reports by school age; and/or older ages at first child protection contacts.

Although we used first-time and re-reporting and investigation patterns in more recently born cohorts of children to estimate the under-ascertainment of child protection contacts in the 2009 AEDC cohort, the numbers of contacts may also have been influenced by policy and reporting practices that changed throughout the study period. Given the overall percent of children with child protection contacts in the 2009 AEDC cohort is similar or higher than more recently born AEDC cohorts, and the patterns of developmental risk in most serious child protection contact groups is similar across all AEDC cycles, it is unlikely that the under-ascertainment of child protection contacts in the 2009 AEDC cohort qualitatively impacted the study findings.

eTable4a. Child protection reports and screened-in reports recorded during and after incomplete data period (2009 AEDC cohort), or equivalent periods (2012-2018 AEDC cohorts) in New South Wales.

	**Children born during incomplete data period or equivalent**		**Children born after incomplete data period**	
**Child protection reports**	**n/N**	**%**	**n/N**	**%**
**Children with AEDC in 2009 (N=93,611)**	**Born before Jan 1 2004[1] (N=62,505; 67%)**		**Born on or after Jan 1 2004 (N=31,106; 33%)**	
≥1 Reports before starting school	9,201/62,505	15%	5,194/31,106	17%
First reported ≤ Dec 31 2003[3]	Incomplete/no data		Incomplete/no data	
Re-Reported ≥ Jan 1 2004	Unable to calculate		Unable to calculate	
**Children with AEDC in 2012 (N=99,778)**	**Born before Jan 1 2007[2] (N=67,739; 68%)**		**Born on or after Jan 1 2007 (N=32,039; 32%)**	
≥1 Reports before starting school	10,634/67,739	16%	5,279/32,039	16%
First reported ≤ Dec 31 2006[3]	3,087/10,634	29%	467/5,279	9%
Re-reported ≥Jan 1 2007	2,492/3,087	81%	434/467	93%
Not re-reported ≥Jan 1 2007[4]	595/3,087	19%	33/467	7%
**Children with AEDC in 2015 (N=101,374)**	**Born before Jan 1 2010[2] (N=71,029; 70%)**		**Born on or after Jan 1 2010 (N=30,345; 30%)**	
≥1 Reports before starting school	9,387/71,029	13%	3,864/30,345	13%
First reported ≤ Dec 31 2009[3]	3,576/9,387	38%	516/3,864	13%
Re-reported ≥ Jan 1 2010	2,630/3,576	74%	454/516	88%
Not re-reported ≥Jan 1 2010[4]	946/3,576	26%	62/516	12%
**Children with AEDC in 2018 (N=103,939)**	**Born before Jan 1 2013[2] (N=75,827; 73%)**		**Born on or after Jan 1 2013 (N=28,112; 27%)**	
≥1 Reports before starting school	9,589/75,827	13%	3,428/28,112	12%
First reported ≤ Dec 31 2012[3]	2,599/9,589	27%	332/3,428	10%
Re-reported ≥Jan 1 2013	2,047/2,599	79%	304/332	92%
Not re-reported ≥Jan 1 2013[4]	552/2,599	21%	28/332	8%
**Screened-in child protection reports**				
**Children with AEDC in 2009 (N=93,611)**	**Born before Jan 1 2004[1] (N=62,505; 67%)**		**Born on or after Jan 1 2004 (N=31,106; 33%)**	
≥1 screened-in reports before school	7,918/62,505	13%	4,420/31,106	14%
First screened-in ≤ Dec 31 2003[3]	Incomplete/no data		Incomplete/no data	
Re-screened-in ≥Jan 1 2004	Unable to calculate		Unable to calculate	
**Children with AEDC in 2012 (N=99,778)**	**Born before Jan 1 2007[2] (N=67,739; 68%)**		**Born on or after Jan 1 2007 (N=32,039; 32%)**	
≥1 screened-in reports starting school	9,008/67,739	13%	4,470/32,039	14%
First screened-in ≤ Dec 31 2006[3]	2,733/9,008	30%	432/4,470	10%
Re-screened-in ≥Jan 1 2007	2,120/2,733	78%	386/432	89%
Not re-screened-in ≥Jan 1 2007[4]	613/2,733	22%	46/432	11%
**Children with AEDC in 2015 (N=101,374)**	**Born before Jan 1 2010[2] (N=71,029; 70%)**		**Born on or after Jan 1 2010 (N=30,345; 30%)**	
≥1 screened-in reports before school	7,492/71,029	11%	2,934/30,345	10%
First screened-in ≤ Dec 31 2009[3]	3,273/7,492	44%	468/2,934	16%
Re-screened-in ≥Jan 1 2010	2,118/3,273	65%	373/468	80%
Not re-screened-in ≥Jan 1 2010[4]	1,155/3,273	35%	95/468	20%
**Children with AEDC in 2018 (N=103,939)**	**Born before Jan 1 2013[2] (N=75,827; 73%)**		**Born on or after Jan 1 2013 (N=28,112; 27%)**	
≥1 screened-in reports before school	7,593/75,827	10%	2,687/28,112	10%
First screened-in ≤Dec 31 2012[3]	1,841/7,593	24%	236/2,687	9%
Re-screened-in ≥ Jan 1 2013	1,332/1,841	72%	208/236	88%
Not re-screened-in ≥Jan 1 2013[4]	509/1,841	28%	28/236	12%

Orange shading highlights children first reported in the incomplete data period equivalent and the numbers not re-reported. Green shading highlights children re-reported after the incomplete data period equivalent, suggesting a similar percent were ascertained for the 2009 AEDC cohort after data were collected. 1. For the 2009 NSW AEDC cohort, this is the period with incomplete data on child protection reports; 2. Children in the 2012-2018 AEDC cohorts born in the incomplete data period equivalent to the 2009 AEDC cohort; 3. For the children born on or after January 1 2004, 2007, 2010, and 2013, the first-reported period include prenatal reports (i.e. before the child was born). 4. The not re-reported groups were used to estimate potential scale of under-ascertainment in the 2009 AEDC cohort.

eTable4b. Investigations and Substantiations recorded during and after incomplete data period (2009 AEDC cohort), or equivalent periods (2012-2018 AEDC cohorts) in New South Wales.

	Children born in incomplete data period or equivalent		Children born after incomplete data period	
**Investigations**	**n/N**	**%**	**n/N**	**%**
**Children with AEDC in 2009 (N=93,611)**	**Born before Jan 1 2005[1] (N=93,011; 99%)**		**Born on or after Jan 1 2005 (N=600; 1%)**	
≥1 investigations before school	2,751/93,011	3%	17/600	3%
First investigated ≤ Dec 31 2004[3]	Incomplete data		Incomplete data	
Re-investigated ≥ Jan 1 2005	Unable to calculate		Unable to calculate	
**Children with AEDC in 2012 (N=99,778)**	**Born before Jan 1 2008[2] (N=99,753; >99%)**		**Born on or after Jan 1 2008 (N=15; <1%)**	
≥1 investigations before school	3,601/99,763	4%	<5/15	-
First investigated ≤ Dec 31 2007[3]	1,584/3,601	44%	Cell sizes too small	
Re-investigated ≥ Jan 1 2008	617/1,584	39%		
Not re-investigated ≥ Jan 1 2008[4]	967/1,584	61%		
**Children with AEDC in 2015 (N=101,374)**	**Born before Jan 1 2011[2] (N=101,359; >99%)**		**Born on or after Jan 1 2011 (N=15; <1%)**	
≥1 investigations before school	3,974/101,359	4%	<5/15	-
First investigated ≤ Dec 31 2010[3]	1,455/3,974	37%	Cell sizes too small	
Re-investigated ≥ Jan 1 2011	571/1,455	39%		
Not re-investigated ≥ Jan 1 2011[4]	884/1,455	61%		
**Children with AEDC in 2018 (N=103,939)**	**Born before Jan 1 2014[2] (N=103,905; >99%)**		**Born on or after Jan 1 2014 (N=34; <1%)**	
≥1 investigations before school	4,969/103,905	5%	<5/34	-
First investigated ≤ Dec 31 2013[3]	1,958/4,969	39%	Cell sizes too small	
Re-investigated ≥ Jan 1 2014	866/1,958	44%		
Not re-investigated ≥ Jan 1 2014[4]	1092/1,958	56%		
**Substantiations**				
**Children with AEDC in 2009 (N=93,611)**	**Born before Jan 1 2005[1] (N=93,011; 99%)**		**Born on or after Jan 1 2005 (N=600; 1%)**	
≥1 substantiations before school	2,416/93,011	3%	15/600	3%
First substantiated ≤ Dec 31 2004[3]	Incomplete data		Cell sizes too small	
Re-substantiated ≥ Jan 1 2005	Unable to calculate			
**Children with AEDC in 2012 (N=99,778)**	**Born before Jan 1 2008[2] (N=99,753; >99%)**		**Born on or after Jan 1 2008 (N=15; <1%)**	
≥1 substantiations before school	3,296/99,763	3%	<5/15	-
First substantiated ≤ Dec 31 2007[3]	1,470/3,296	45%	Cell sizes too small	
Re-substantiated ≥ Jan 1 2008	532/1,470	36%		
Not re-substantiated ≥ Jan 1 2008[4]	938/1,470	64%		
**Children with AEDC in 2015 (N=101,374)**	**Born before Jan 1 2011[2] (N=101,359; >99%)**		**Born on or after Jan 1 2011 (N=15; <1%)**	
≥1 substantiations before school	3,405/101,359	3%	<5/15	-
First substantiated ≤ Dec 31 2010[3]	1,331/3,405	39%	Cell sizes too small	
Re-substantiated ≥ Jan 1 2011	466/1,331	35%		
Not re-substantiated ≥ Jan 1 2011[4]	865/1,331	65%		
**Children with AEDC in 2018 (N=103,939)**	**Born before Jan 1 2014[2] (N=103,905; >99%)**		**Born on or after Jan 1 2014 (N=34; <1%)**	
≥1 substantiations before school	3,687/103,905	4%	<5/34	-
First substantiated ≤ Dec 31 2013[3]	1,698/3,687	46%	Cell sizes too small	
Re-substantiated ≥ Jan 1 2014	562/1,698	33%		
Not re-substantiated ≥ Jan 1 2014[4]	1,136/1,698	67%		

Orange shading highlights children first reported in the incomplete data period equivalent and the numbers not re-reported. Green shading highlights children re-reported after the incomplete data period equivalent, suggesting a similar percent were ascertained for the 2009 AEDC cohort after data were collected. 1. For the 2009 NSW AEDC cohort, this is the period with incomplete data on investigations; 2. Children in the 2012-2018 AEDC cohorts born in the incomplete data period equivalent to the 2009 AEDC cohort; 3. For the children born on or after January 1 2005, 2008, 2011, and 2014, the first-reported period include prenatal investigations (i.e. before the child was born). 4. The not re-investigated groups were used to estimate potential scale of under-ascertainment in the 2009 AEDC cohort.

eTable4c. Adjusted 2009 AEDC cohort estimates for numbers and percent of children with child protection contacts, using re-reporting and investigation numbers from the 2012-2018 AEDC cohorts in New South Wales (reported in eTables 4a and 4b).

	**Children born in incomplete data period**				**Children born after incomplete data period**			
	Child protection contact before school in 2009 AEDC cohort, N[1]	Not re-recorded in 2012-2018 AEDC cohorts, N[1]	Adjusted 2009, N	Adjusted 2009, %	Number with child protection contact before school in 2009 AEDC cohort[1]	Not re-recorded number from 2012-2018 AEDC cohorts[1]	Adjusted 2009 number	Adjusted 2009 %
Child protection reports								
2012 AEDC based estimates	9201	595	9796	16%	5194	33	5227	17%
2015 AEDC based estimates	9201	946	10147	16%	5194	62	5256	17%
2018 AEDC based estimates	9201	552	9753	16%	5194	28	5222	17%
Screened-in child protection reports								
2012 AEDC based estimates	7918	613	8531	14%	4420	46	4466	14%
2015 AEDC based estimates	7918	1155	9073	15%	4420	95	4515	15%
2018 AEDC based estimates	7918	509	8427	13%	4420	28	4448	14%
Investigations								
2012 AEDC based estimates	2751	967	3718	4%				
2015 AEDC based estimates	2751	884	3635	4%				
2018 AEDC based estimates	2751	1092	3843	4%				
Substantiations								
2012 AEDC based estimates	2416	938	3354	4%				
2015 AEDC based estimates	2416	865	3281	4%				
2018 AEDC based estimates	2416	1136	3552	4%				

AEDC, Australian Early Development Census. 1. Reported in Tables S4a and S4b in Supplement 4.

eTable5 Demographic characteristics by most serious type of child protection contact before school entry, in New South Wales and South Australia, all AEDC years combined

	New South Wales							South Australia						
	Most serious type of child protection contact before school entry							Most serious type of child protection contact before school entry						
	No contact<br>n<br>(Col %)	Reported<br>n<br>(Col %)	Screened-in<br>n<br>(Col %)	Investigated<br>n<br>(Col %)	Substantiated<br>n<br>(Col %)	OOHC<br>n<br>(Col %)	Total<br>N<br>(Col %)	No contact<br>n<br>(Col %)	Reported<br>n<br>(Col %)	Screened-in<br>n<br>(Col %)	Investigated<br>n<br>(Col %)	Substantiated<br>n<br>(Col %)	OOHC<br>n<br>(Col %)	Total<br>N<br>(Col %)
**Child's sex**														
Male	175,415<br>(51.3)	5,279<br>(52.9)	16,150<br>(52.4)	1,135<br>(49.7)	4,149<br>(50.8)	2,779<br>(51.7)	204,907<br>(51.4)	35,087<br>(51.5)	2,109<br>(51.2)	2,254<br>(52.0)	859<br>(51.4)	824<br>(53.4)	485<br>(51.4)	41,618<br>(51.6)
Female	166,637<br>(48.7)	4,707<br>(47.1)	14,698<br>(47.6)	1,148<br>(50.3)	4,012<br>(49.2)	2,593<br>(48.3)	193,795<br>(48.6)	33,027<br>(48.5)	2,012<br>(48.8)	2,082<br>(48.0)	813<br>(48.6)	720<br>(46.6)	459<br>(48.6)	39,113<br>(48.4)
**Aboriginal and/or Torres Strait Islander children (First Nations)[1]**														
No	324,428<br>(94.8)	8,408<br>(84.2)	23,744<br>(77.0)	1,573<br>(68.9)	5,118<br>(62.7)	2,869<br>(53.4)	366,140<br>(91.8)	65,778<br>(96.6)	3,620<br>(87.8)	3,489<br>(80.5)	1,231<br>(73.6)	1042<br>(67.5)	577<br>(61.1)	75,737<br>(93.8)
Yes	17,624<br>(5.2)	1,578<br>(15.8)	7,104<br>(23.0)	710<br>(31.1)	3,043<br>(37.3)	2,503<br>(46.6)	32,562<br>(8.2)	2,336<br>(3.4)	501<br>(12.2)	847<br>(19.5)	441<br>(26.4)	502<br>(32.5)	367<br>(38.9)	49,94<br>(6.2)
**Mother's age at childbirth[2]**														
<20 years	5,828<br>(1.7)	753<br>(7.5)	3,200<br>(10.4)	246<br>(10.8)	1,099<br>(13.5)	783<br>(14.6)	11,909<br>(3.0)	1,410<br>(2.1)	399<br>(9.7)	509<br>(11.7)	303<br>(18.1)	232<br>(15.0)	171<br>(18.1)	3,024<br>(3.7)
≥20 years	285,911<br>(83.6)	8,096<br>(81.1)	24,730<br>(80.2)	1,753<br>(76.8)	6,153<br>(75.4)	4,046<br>(75.3)	330,689<br>(82.9)	57,115<br>(83.9)	3,191<br>(77.4)	3,350<br>(77.3)	1,217<br>(72.8)	1,158<br>(75.0)	675<br>(71.5)	66,706<br>(82.6)
Not available	50,313<br>(14.7)	1,137<br>(11.4)	2,918<br>(9.5)	284<br>(12.4)	909<br>(11.1)	543<br>(10.1)	56,104<br>(14.1)	9,589<br>(14.1)	531<br>(12.9)	477<br>(11.0)	152<br>(9.1)	154<br>(10.0)	98<br>(10.4)	11,001<br>(13.6)
**Other parent's age at childbirth[3]**														
<20 years	1,333<br>(0.4)	200<br>(2.0)	861<br>(2.8)	66<br>(2.9)	285<br>(3.5)	158<br>(2.9)	2,903<br>(0.7)	474<br>(0.7)	152<br>(3.7)	176<br>(4.1)	102<br>(6.1)	91<br>(5.9)	51<br>(5.4)	1,046<br>(1.3)
≥20 years	284,041<br>(83.0)	7,994<br>(80.1)	24,133<br>(78.2)	1,629<br>(71.4)	5,683<br>(69.6)	3,218<br>(59.9)	326,698<br>(81.9)	56,833<br>(83.4)	3,180<br>(77.2)	3,258<br>(75.1)	1,182<br>(70.7)	1,061<br>(68.7)	552<br>(58.5)	66,066<br>(81.8)
Not available	56,678<br>(16.6)	1,792<br>(17.9)	5,854<br>(19.0)	588<br>(25.8)	2,193<br>(26.9)	1,996<br>(37.2)	69,101<br>(17.3)	10,807<br>(15.9)	789<br>(19.1)	902<br>(20.8)	388<br>(23.2)	392<br>(25.4)	341<br>(36.1)	13,619<br>(16.9)
**Mother married/partnered at childbirth[4,5]**														
No	32,517<br>(9.5)	2,843<br>(28.5)	10,951<br>(35.5)	872<br>(38.2)	3,601<br>(44.1)	3,007<br>(56.0)	53,791<br>(13.5)	4,858<br>(7.1)	823<br>(20.0)	1135<br>(26.2)	526<br>(31.5)	513<br>(33.2)	419<br>(44.4)	8,274<br>(10.2)
Yes	252,649<br>(73.9)	5,761<br>(57.7)	16,145<br>(52.3)	1,077<br>(47.2)	3,445<br>(42.2)	1,647<br>(30.7)	280,724<br>(70.4)	53,501<br>(78.5)	2757<br>(66.9)	2716<br>(62.6)	989<br>(59.2)	875<br>(56.7)	423<br>(44.8)	61,261<br>(75.9)
Not available	56,886<br>(16.6)	1,382<br>(13.8)	3,752<br>(12.2)	334<br>(14.6)	1,115<br>(13.7)	718<br>(13.4)	64,187<br>(16.1)	9,755<br>(14.3)	541<br>(13.1)	485<br>(11.2)	157<br>(9.4)	156<br>(10.1)	102<br>(10.8)	11,196<br>(13.9)

	New South Wales							South Australia						
	Most serious type of child protection contact before school entry							Most serious type of child protection contact before school entry						
	No contact n (Col %)	Reported n (Col %)	Screened-in n (Col %)	Investigated n (Col %)	Substantiated n (Col %)	OOHC n (Col %)	Total N (Col %)	No contact n (Col %)	Reported n (Col %)	Screened-in n (Col %)	Investigated n (Col %)	Substantiated n (Col %)	OOHC n (Col %)	Total N (Col %)
**Mother's country of birth[4,5]**														
Australia	201,030 (58.8)	6,576 (65.9)	21,956 (71.2)	1,652 (72.4)	6,198 (75.9)	4,325 (80.5)	241,737 (60.6)	47,486 (69.7)	3,095 (75.1)	3,366 (77.6)	1,387 (83.0)	1293 (83.7)	794 (84.1)	57,421 (71.1)
Other	89,526 (26.2)	2,245 (22.5)	5,869 (19.0)	334 (14.6)	1,019 (12.5)	462 (8.6)	99,455 (24.9)	10,884 (16)	487 (11.8)	487 (11.2)	129 (7.7)	96 (6.2)	48 (5.1)	12,131 (15.0)
Not available	51,496 (15.1)	1,165 (11.7)	3,023 (9.8)	297 (13.0)	944 (11.6)	585 (10.9)	57,510 (14.4)	9,744 (14.3)	539 (13.1)	483 (11.1)	156 (9.3)	155 (10.0)	102 (10.8)	11,179 (13.8)
**Private/public patient at child's birth[4,5]**														
Public	180,437 (52.8)	7,233 (72.4)	24,285 (78.7)	1,795 (78.6)	6,762 (82.9)	4,670 (86.9)	225,182 (56.5)	37,578 (55.2)	3,115 (75.6)	3,553 (81.9)	1,432 (85.6)	1,323 (85.7)	829 (87.8)	47,830 (59.2)
Private	27,869 (8.1)	478 (4.8)	1,058 (3.4)	84 (3.7)	165 (2.0)	43 (0.8)	29,697 (7.4)	20,792 (30.5)	467 (11.3)	300 (6.9)	84 (5.0)	66 (4.3)	14 (1.5)	21,723 (26.9)
Not available	133,746 (39.1)	2,275 (22.8)	5,505 (17.8)	404 (17.7)	1,234 (15.1)	659 (12.3)	143,823 (36.1)	9,744 (14.3)	539 (13.1)	483 (11.1)	156 (9.3)	155 (10)	101 (10.7)	11,178 (13.8)
**Area level disadvantage of residence at child's birth[6]**														
Quintile 1 (most disadvantaged)	46,044 (13.5)	2,071 (20.7)	6,643 (21.5)	669 (29.3)	2,245 (27.5)	1,441 (26.8)	59,113 (14.8)	14,280 (21.0)	1,439 (34.9)	1,797 (41.4)	770 (46.1)	735 (47.6)	488 (51.7)	19,509 (24.2)
Quintile 2	41,553 (12.1)	1,610 (16.1)	4,884 (15.8)	445 (19.5)	1,703 (20.9)	1,014 (18.9)	51,209 (12.8)	14,002 (20.6)	947 (23.0)	969 (22.3)	357 (21.4)	353 (22.9)	181 (19.2)	16,809 (20.8)
Quintile 3	78,724 (23.0)	2,630 (26.3)	9,282 (30.1)	489 (21.4)	2,042 (25.0)	1,471 (27.4)	94,638 (23.7)	9,625 (14.1)	457 (11.1)	453 (10.4)	181 (10.8)	153 (9.9)	88 (9.3)	10,957 (13.6)
Quintile 4	51,231 (15.0)	1,288 (12.9)	3,990 (12.9)	216 (9.5)	723 (8.9)	575 (10.7)	58,023 (14.6)	12,047 (17.7)	472 (11.5)	413 (9.5)	147 (8.8)	97 (6.3)	62 (6.6)	13,238 (16.4)
Quintile 5 (most advantaged)	74,210 (21.7)	1,256 (12.6)	3,165 (10.3)	192 (8.4)	560 (6.9)	328 (6.1)	79,711 (20.0)	8,518 (12.5)	272 (6.6)	224 (5.2)	65 (3.9)	50 (3.2)	27 (2.9)	9,156 (11.3)
Not available	50,290 (14.7)	1,131 (11.3)	2,884 (9.3)	272 (11.9)	888 (10.9)	543 (10.1)	56,008 (14.0)	9,642 (14.2)	534 (13.0)	480 (11.1)	152 (9.1)	156 (10.1)	98 (10.4)	11,062 (13.7)
**Geographical remoteness of residence at child's birth[7]**														
Major Cities	229,106 (67.0)	6,503 (65.1)	19,129 (62.0)	1,218 (53.4)	4,157 (50.9)	3,087 (57.5)	263,200 (66.0)	42,989 (63.1)	2,534 (61.5)	2,940 (67.8)	972 (58.1)	836 (54.1)	564 (59.7)	50,835 (63.0)
Inner Regional	44,520 (13.0)	1,698 (17.0)	6,371 (20.7)	551 (24.1)	2,175 (26.7)	1,236 (23.0)	56,551 (14.2)	6,822 (10.0)	456 (11.1)	350 (8.1)	206 (12.3)	181 (11.7)	97 (10.3)	8,112 (10.0)
Outer Regional	14,425 (4.2)	555 (5.6)	2,133 (6.9)	212 (9.3)	820 (10.0)	444 (8.3)	18,589 (4.7)	6,386 (9.4)	478 (11.6)	469 (10.8)	283 (16.9)	285 (18.5)	136 (14.4)	8,037 (10.0)

	New South Wales							South Australia						
	Most serious type of child protection contact before school entry							Most serious type of child protection contact before school entry						
	**No contact n (Col %)**	**Reported n (Col %)**	**Screened-in n (Col %)**	**Investigated n (Col %)**	**Substantiated n (Col %)**	**OOHC n (Col %)**	**Total N (Col %)**	**No contact n (Col %)**	**Reported n (Col %)**	**Screened-in n (Col %)**	**Investigated n (Col %)**	**Substantiated n (Col %)**	**OOHC n (Col %)**	**Total N (Col %)**
Remote/Very Remote	1,217 (0.4)	58 (0.6)	273 (0.9)	28 (1.2)	108 (1.3)	51 (0.9)	1,735 (0.4)	2,327 (3.4)	122 (3.0)	100 (2.3)	59 (3.5)	88 (5.7)	49 (5.2)	2,745 (3.4)
Not available	52,784 (15.4)	1,172 (11.7)	2,942 (9.5)	274 (12.0)	901 (11.0)	554 (10.3)	58,627 (14.7)	9,590 (14.1)	531 (12.9)	477 (11.0)	152 (9.1)	154 (10.0)	98 (10.4)	11,002 (13.6)
**Age at AEDC[8]**														
<5 years 1 month	26,190 (7.7)	813 (8.1)	2,696 (8.7)	189 (8.3)	659 (8.1)	297 (5.5)	30,844 (7.7)	2,157 (3.2)	129 (3.1)	110 (2.5)	56 (3.3)	55 (3.6)	20 (2.1)	2,527 (3.1)
≥5 y 1 month & <5 y 4 months	54,124 (15.8)	1,666 (16.7)	5,032 (16.3)	362 (15.9)	1,270 (15.6)	703 (13.1)	63,157 (15.8)	12,836 (18.8)	747 (18.1)	754 (17.4)	271 (16.2)	258 (16.7)	164 (17.4)	15,030 (18.6)
≥5 y 4 months & < 5 y 7 months	76,176 (22.3)	2,132 (21.3)	6,564 (21.3)	480 (21.0)	1,665 (20.4)	1,057 (19.7)	88,074 (22.1)	16,590 (24.4)	969 (23.5)	1,066 (24.6)	413 (24.7)	368 (23.8)	204 (21.6)	19,610 (24.3)
≥5 y 7 months & <5 y 10 months	83,906 (24.5)	2,332 (23.4)	7,183 (23.3)	482 (21.1)	1,851 (22.7)	1,232 (22.9)	96,986 (24.3)	17,450 (25.6)	1042 (25.3)	1,028 (23.7)	424 (25.4)	363 (23.5)	213 (22.6)	20,520 (25.4)
≥5 y 10 months & < 6 y 1 month	62,248 (18.2)	1,806 (18.1)	5,447 (17.7)	422 (18.5)	1,504 (18.4)	1,093 (20.3)	72,520 (18.2)	14,682 (21.6)	901 (21.9)	1,015 (23.4)	346 (20.7)	337 (21.8)	212 (22.5)	17,493 (21.7)
≥ 6 y 1 month	39,408 (11.5)	1,237 (12.4)	3,926 (12.7)	348 (15.2)	1,212 (14.9)	990 (18.4)	47,121 (11.8)	4,399 (6.5)	333 (8.1)	363 (8.4)	162 (9.7)	163 (10.6)	131 (13.9)	5,551 (6.9)
**Child speaks English as a second language[8]**														
No	273,009 (79.8)	8,362 (83.7)	26,555 (86.1)	2,056 (90.1)	7,430 (91.0)	5,072 (94.4)	322,484 (80.9)	58,143 (85.4)	3,653 (88.6)	3,793 (87.5)	1,505 (90.0)	1,335 (86.5)	841 (89.1)	69,270 (85.8)
Yes	69,043 (20.2)	1,624 (16.3)	4,293 (13.9)	227 (9.9)	731 (9.0)	300 (5.6)	76,218 (19.1)	9,971 (14.6)	468 (11.4)	543 (12.5)	167 (10.0)	209 (13.5)	103 (10.9)	11,461 (14.2)
**Maternal year of high school completed[9]**														
Year 9 or equivalent, or below	9,926 (4.1)	702 (8.7)	3,400 (12.8)	299 (15.4)	1,369 (19.3)	482 (13.8)	16,178 (5.5)							
Year 10 or equivalent	41,214 (16.8)	2,216 (27.4)	8,318 (31.4)	617 (31.8)	2,284 (32.1)	656 (18.8)	55,305 (19.0)							
Year 11 or equivalent	15,203 (6.2)	850 (10.5)	2,826 (10.7)	180 (9.3)	724 (10.2)	196 (5.6)	19,979 (6.8)							
Year 12 or equivalent	174,486 (71.3)	4,035 (49.8)	10,301 (38.9)	690 (35.6)	1,862 (26.2)	515 (14.8)	191,889 (65.8)							
Not available	3,907 (1.6)	297 (3.7)	1,628 (6.1)	154 (7.9)	872 (12.3)	1,637 (47.0)	8,495 (2.9)							

	New South Wales							South Australia						
	Most serious type of child protection contact before school entry							Most serious type of child protection contact before school entry						
	No contact n (Col %)	Reported n (Col %)	Screened-in n (Col %)	Investigated n (Col %)	Substantiated n (Col %)	OOHC n (Col %)	Total N (Col %)	No contact n (Col %)	Reported n (Col %)	Screened-in n (Col %)	Investigated n (Col %)	Substantiated n (Col %)	OOHC n (Col %)	Total N (Col %)
**Highest parental education[10]**														
No post-school qualification								9,041 (13.3)	1,130 (27.4)	1,561 (36.0)	662 (39.6)	702 (45.5)	269 (28.5)	13,365 (16.6)
Certificate/ Diploma								21,100 (31.0)	1,707 (41.4)	1,593 (36.7)	525 (31.4)	431 (27.9)	232 (24.6)	25,588 (31.7)
Bachelor degree or above								20,521 (30.1)	575 (14.0)	374 (8.6)	102 (6.1)	70 (4.5)	93 (9.9)	21,735 (26.9)
Not available								17,452 (25.6)	709 (17.2)	808 (18.6)	383 (22.9)	341 (22.1)	350 (37.1)	20,043 (24.8)
**Parental employment[9,10]**														
≥1 parent in the labour force	229,428 (67.1)	6,628 (66.4)	19,448 (63.0)	1,237 (54.2)	3,959 (48.5)	1,105 (20.6)	261,805 (65.7)	55,392 (81.3)	2,975 (72.2)	2,798 (64.5)	964 (57.7)	786 (50.9)	309 (32.7)	63,224 (78.3)
No parent in the labour force	12,399 (3.6)	1,318 (13.2)	6,096 (19.8)	627 (27.5)	2,683 (32.9)	1,063 (19.8)	24,186 (6.1)	3,127 (4.6)	615 (14.9)	1,061 (24.5)	555 (33.2)	604 (39.1)	536 (56.8)	6498 (8.0)
Not available	100,225 (29.3)	2,040 (20.4)	5,304 (17.2)	419 (18.4)	1,519 (18.6)	3,204 (59.6)	112,711 (28.3)	9,595 (14.1)	531 (12.9)	477 (11)	153 (9.2)	154 (10)	99 (10.5)	11,009 (13.6)
**Area level disadvantage of residence in first-year of full-time school[8]**														
Quintile 1 (most disadvantaged)	64,973 (19.0)	3,166 (31.8)	11,869 (38.6)	970 (42.6)	4,016 (49.3)	2,080 (38.8)	87,074 (21.9)	15,400 (22.6)	1,739 (42.2)	2,217 (51.1)	904 (54.1)	906 (58.7)	455 (48.2)	21621 (26.8)
Quintile 2	63,207 (18.5)	2,333 (23.4)	7,163 (23.3)	532 (23.4)	1,748 (21.5)	1,308 (24.4)	76,291 (19.2)	16,652 (24.4)	1,025 (24.9)	1050 (24.2)	390 (23.3)	325 (21.0)	236 (25.0)	19678 (24.4)
Quintile 3	61,271 (17.9)	1,777 (17.8)	4,916 (16.0)	331 (14.5)	1,117 (13.7)	889 (16.6)	70,301 (17.7)	15,308 (22.5)	743 (18.0)	592 (13.7)	207 (12.4)	194 (12.6)	141 (14.9)	17185 (21.3)
Quintile 4	62,829 (18.4)	1,371 (13.8)	3,653 (11.9)	266 (11.7)	770 (9.5)	656 (12.2)	69,545 (17.5)	13,076 (19.2)	443 (10.7)	338 (7.8)	125 (7.5)	77 (5.0)	82 (8.7)	14141 (17.5)
Quintile 5 (most advantaged)	89,158 (26.1)	1,318 (13.2)	3,179 (10.3)	177 (7.8)	490 (6.0)	430 (8.0)	94,752 (23.8)	7,539 (11.1)	165 (4.0)	133 (3.1)	38 (2.3)	31 (2.0)	22 (2.3)	7928 (9.8)
Not available								139 (0.2)	6 (0.1)	6 (0.1)	8 (0.5)	11 (0.7)	8 (0.8)	178 (0.2)
**Geographical remoteness of residence in first-year of full-time school[8]**														
Major Cities	259,615 (75.9)	7,040 (70.5)	20,342 (65.9)	1,300 (56.9)	4,441 (54.4)	3,121 (58.1)	295,859 (74.2)	48,973 (71.9)	2821 (68.5)	3167 (73)	996 (59.6)	839 (54.3)	558 (59.1)	57354 (71.0)
Inner Regional	60,244 (17.6)	2,157 (21.6)	7,534 (24.4)	695 (30.4)	2,552 (31.3)	1,601 (29.8)	74,783 (18.8)	8,982 (13.2)	567 (13.8)	476 (11)	264 (15.8)	240 (15.5)	158 (16.7)	10687 (13.2)

	New South Wales							South Australia						
	Most serious type of child protection contact before school entry							Most serious type of child protection contact before school entry						
	No contact n (Col %)	Reported n (Col %)	Screened-in n (Col %)	Investigated n (Col %)	Substantiated n (Col %)	OOHC n (Col %)	Total N (Col %)	No contact n (Col %)	Reported n (Col %)	Screened-in n (Col %)	Investigated n (Col %)	Substantiated n (Col %)	OOHC n (Col %)	Total N (Col %)
Outer Regional	19,875 (5.8)	702 (7.0)	2,617 (8.5)	261 (11.4)	1,039 (12.7)	580 (10.8)	25,074 (6.3)	7,430 (10.9)	543 (13.2)	542 (12.5)	323 (19.3)	334 (21.6)	169 (17.9)	9341 (11.6)
Remote/Very Remote [11]	2,318 (0.7)	87 (0.9)	355 (1.2)	27 (1.2)	129 (1.6)	70 (1.3)	2,986 (0.7)	2,729 (4.0)	190 (4.6)	151 (3.5)	89 (5.3)	131 (8.5)	59 (6.3)	3349 (4.1)
**Total**	**342,052 (100.0)**	**9,986 (100.0)**	**30,848 (100.0)**	**2,283 (100.0)**	**8,161 (100.0)**	**5,372 (100.0)**	**398,702 (100.0)**	**68,114 (100.0)**	**4,121 (100.0)**	**4,336 (100.0)**	**1,672 (100.0)**	**1,544 (100.0)**	**944 (100.0)**	**80,731 (100.0)**

AEDC, Australian Early Development Census; OOHC, Out-of-home care; Not available, includes missing data and no linked records for the relevant data source. 1. Child, mother and/or other parent had one or more birth, child protection, AEDC or school enrolment records with Aboriginal and/or Torres Strait Islander recorded; 2. Mother's age at childbirth was defined using mother's and child's dates of birth in the perinatal, birth registration and/or hospital records at birth; 3. Other parent's age at childbirth was defined using other parent's and child's dates of birth recorded in birth registration; 4. Obtained from the NSW hospital birth record; 5. Obtained from the SA perinatal record; 6. Based on the Index of Relative Socio-economic Advantage and Disadvantage for the Statistical Area 2, Statistical Local Area and postcode of residence at the child's birth in the perinatal, birth registration and/or hospital birth record or AEDC; 7. Based on the Accessibility/Remoteness Index of Australia (ARIA+) for the Statistical Area 2,Statistical Local Area and postcode of residence at the child's birth in the perinatal, birth registration and/or hospital birth; 8. Obtained from AEDC record; 9. Recorded on the Public School Enrolment record in NSW; 10. Recorded on the child's birth registration in SA; 11. In NSW, there were <5 missing values for the Geographical remoteness of residence in the first-year of full-time school variable, which have been included in the Remote/Very Remote category total to suppress small cell sizes.

eFigure4 The risk of developmental vulnerability on ≥1 domains[1] or medically diagnosed conditions2 at school entry, by number of child protection reports before school, among New South Wales and South Australian children in their first year of full-time school (all AEDC years combined[1]).

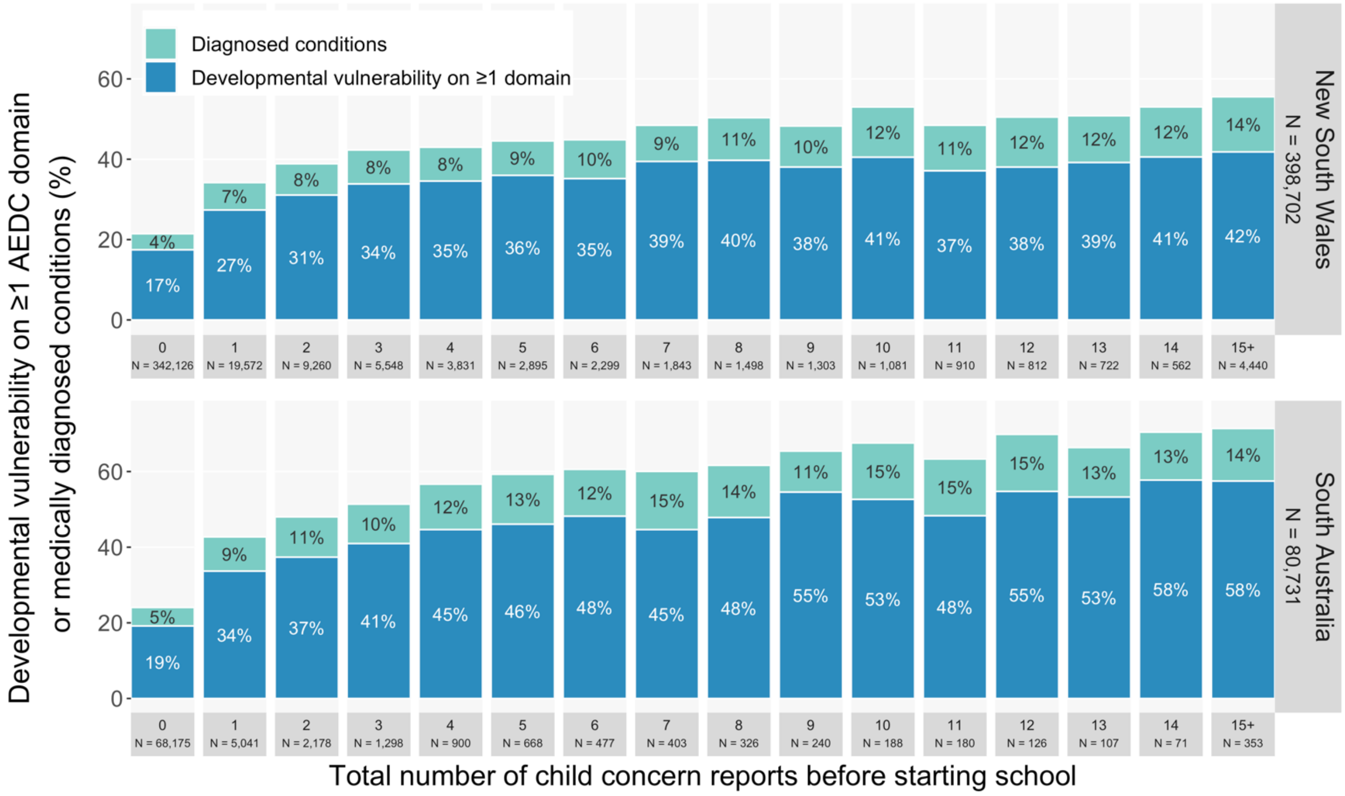

AEDC, Australian Early Development Census; NSW, New South Wales. 1. 2009, 2012, 2015 and 2018 AEDC cycles. 1. The AEDC DV1 summary indicator was used for the outcome 'developmental vulnerability on ≥1 AEDC domains'; 2. Medically diagnosed conditions with already identified substantial developmental support needs, as listed in eTable 2.

eTable6 The risk of developmental vulnerability on ≥1 domain[1] or medically diagnosed conditions[2] at school entry, by number of child protection reports before school, among New South Wales and South Australia children in their first year of full-time school (all AEDC years combined[1]) (data table for eFigure4).

Total number of child concern reports before starting school	New South Wales							
	Developmental vulnerability on ≥1 domain or medically diagnosed conditions						Total	
	No		Developmental vulnerability on ≥1 domain		Diagnosed conditions			
	n	row %	n	row %	n	row %	n	row %
None	268,714	78.5	59,781	17.5	13,631	4.0	342,126	100.0
1	12,882	65.8	5,359	27.4	1331	6.8	19,572	100.0
2	5,660	61.1	2878	31.1	722	7.8	9,260	100.0
3	3201	57.7	1879	33.9	468	8.4	5,548	100.0
4	2184	57.0	1324	34.6	323	8.4	3831	100.0
5	1605	55.4	1042	36.0	248	8.6	2895	100.0
6	1268	55.2	809	35.2	222	9.7	2299	100.0
7	950	51.5	727	39.4	166	9.0	1843	100.0
8	744	49.7	595	39.7	159	10.6	1498	100.0
9	674	51.7	496	38.1	133	10.2	1303	100.0
10	508	47.0	438	40.5	135	12.5	1081	100.0
11	469	51.5	338	37.1	103	11.3	910	100.0
12	402	49.5	309	38.1	101	12.4	812	100.0
13	355	49.2	283	39.2	84	11.6	722	100.0
14	264	47.0	228	40.6	70	12.5	562	100.0
15+	1974	44.5	1857	41.8	609	13.7	4440	100.0
**Total**	**301,854**	**75.7**	**78,343**	**19.6**	**18,505**	**4.6**	**398,702**	**100.0**
	**South Australia**							
None	51,766	75.9	13,080	19.2	3,329	4.9	68,175	100.0
1	2,887	57.3	1,697	33.7	457	9.1	5,041	100.0
2	1,132	52.0	814	37.4	232	10.7	2,178	100.0
3	631	48.6	532	41.0	135	10.4	1,298	100.0
4	390	43.3	402	44.7	108	12.0	900	100.0
5	272	40.7	308	46.1	88	13.2	668	100.0
6	188	39.4	230	48.2	59	12.4	477	100.0
7	161	40.0	180	44.7	62	15.4	403	100.0
8	125	38.3	156	47.9	45	13.8	326	100.0
9	83	34.6	131	54.6	26	10.8	240	100.0
10	61	32.4	99	52.7	28	14.9	188	100.0
11	66	36.7	87	48.3	27	15.0	180	100.0
12	38	30.2	69	54.8	19	15.1	126	100.0
13	36	33.6	57	53.3	14	13.1	107	100.0
14	21	29.6	41	57.7	9	12.7	71	100.0
15+	101	28.6	203	57.5	49	13.9	353	100
**Total**	**57,958**	**71.8**	**18,086**	**22.4**	**4,687**	**5.8**	**80,731**	**100**

AEDC, Australian Early Development Census; NSW, New South Wales; SA, South Australian. 1. The AEDC DV1 summary indicator was used for the outcome 'developmental vulnerability on ≥1 AEDC domains'; 2. Medically diagnosed conditions with already identified substantial developmental support needs, as listed in eTable 2.

eFigure5 The risk of developmental vulnerability on ≥1 domain[1] or medically diagnosed conditions[2] at school entry, by age at first child protection contact and number of child protection reports before school, among New South Wales and South Australian children in their first year of full-time school (all AEDC years combined[1]).

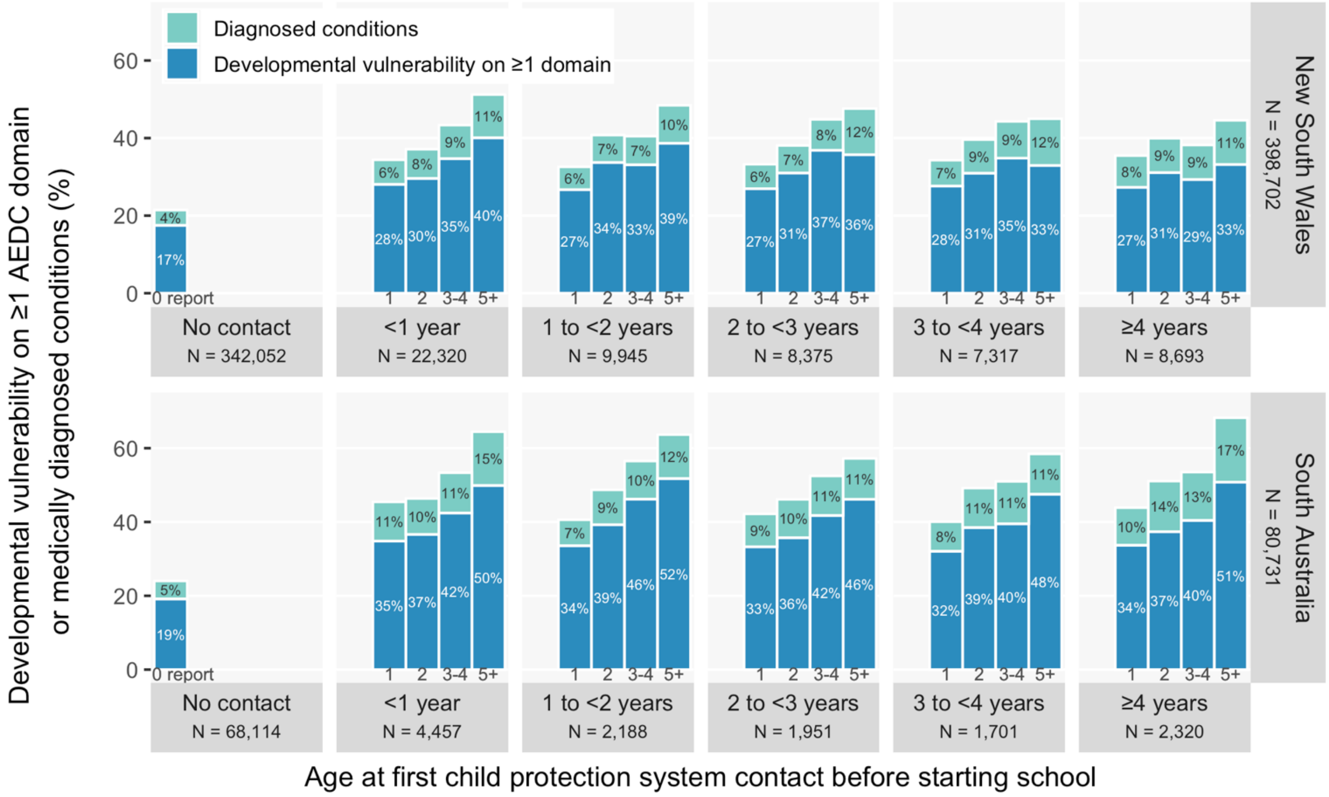

AEDC, Australian Early Development Census; NSW, New South Wales; SA, South Australian. 1. AEDC years include 2009, 2012, 2015 and 2018. 1. The AEDC DV1 summary indicator was used for the outcome 'developmental vulnerability on ≥1 AEDC domains'; 2. Medically diagnosed conditions with already identified substantial developmental support needs, as listed in eTable 2.

eTable7 The risk of developmental vulnerability on ≥1 domain[1] or medically diagnosed conditions[2] at age five, by age at first child protection contact and number of child protection reports before school, among New South Wales and South Australian children in their first year of school (all AEDC years combined[3]) (data table for eFigure5).

Age at first child protection system contact and number of reports before starting school	New South Wales							
	Risk of developmental vulnerability on ≥1 domain or medically diagnosed conditions						Total	
	No		Developmental vulnerability on ≥1 domain		Diagnosed conditions			
	n	row %	n	row %	n	row %	n	row %
**No contact**	**268,667**	**78.5**	**59,767**	**17.5**	**13,618**	**4.0**	**342,052**	**100.0**
**<1 year**	**12,242**	**54.8**	**7,976**	**35.7**	**2102**	**9.4**	**22,320**	**100.0**
1 report	2571	65.6	1100	28.1	248	6.3	3919	100.0
2 reports	1754	62.9	826	29.6	210	7.5	2790	100.0
3-4 reports	2184	56.7	1338	34.7	333	8.6	3855	100.0
5+ reports	5733	48.8	4712	40.1	1311	11.2	11,756	100.0
**1 to <2 years**	**5,914**	**59.5**	**3272**	**32.9**	**759**	**7.6**	**9,945**	**100.0**
1 report	2168	67.4	858	26.7	190	5.9	3216	100.0
2 reports	1018	59.2	579	33.7	122	7.1	1719	100.0
3-4 reports	1088	59.5	605	33.1	135	7.4	1828	**100.0**
5+ reports	1640	51.5	1230	38.7	312	9.8	3182	**100.0**
**2 to <3 years**	**5,082**	**60.7**	**2625**	**31.3**	**668**	**8.0**	**8,375**	100.0
1 report	2314	66.7	934	26.9	219	6.3	3467	100.0
2 reports	1017	61.9	509	31.0	117	7.1	1643	100.0
3-4 reports	806	55.2	538	36.8	117	8.0	1461	100.0
5+ reports	945	52.4	644	35.7	215	11.9	1804	**100.0**
**3 to <4 years**	**4492**	**61.4**	**2215**	**30.3**	**610**	**8.3**	**7,317**	100.0
1 report	2335	65.7	982	27.6	237	6.7	3554	100.0
2 reports	884	60.4	453	30.9	127	8.7	1464	100.0
3-4 reports	675	55.7	422	34.8	115	9.5	1212	**100.0**
5+ reports	598	55.0	358	32.9	131	12.1	1087	**100.0**
**4 years until starting school**	**5,457**	**62.8**	**2488**	**28.6**	**748**	**8.6**	**8,693**	100.0
1 report	3541	64.5	1499	27.3	450	8.2	5,490	100.0
2 reports	987	60.0	511	31.1	146	8.9	1644	100.0
3-4 reports	632	61.8	300	29.3	91	8.9	1023	100.0
5+ reports	297	55.4	178	33.2	61	11.4	536	**100.0**
**Total**	**301,854**	**75.7**	**78,343**	**19.6**	**18,505**	**4.6**	**398,702**	100.0
	South Australia							
**No contact**	**51,735**	**76**	**13,065**	**19.2**	**3,314**	**4.9**	**68,114**	**100.0**
**<1 year**	**1,982**	**44.5**	**1,926**	**43.2**	**549**	**12.3**	**4,457**	**100.0**
1 report	534	54.5	341	34.8	104	10.6	979	100.0
2 reports	341	53.6	233	36.6	62	9.7	636	100.0
3-4 reports	409	46.6	372	42.4	96	10.9	877	100.0
5+ reports	698	35.5	980	49.9	287	14.6	1,965	100.0
**1 to <2 years**	**1,058**	**48.4**	**922**	**42.1**	**208**	**9.5**	**2,188**	**100.0**
1 report	446	59.4	252	33.6	53	7.1	751	100.0
2 reports	200	51.3	153	39.2	37	9.5	390	100.0
3-4 reports	193	43.5	205	46.2	46	10.4	444	100.0
5+ reports	219	36.3	312	51.7	72	11.9	603	100.0
**2 to <3 years**	**1,023**	**52.4**	**734**	**37.6**	**194**	**9.9**	**1,951**	**100.0**
1 report	479	57.8	276	33.3	74	8.9	829	**100.0**
2 reports	223	53.9	148	35.7	43	10.4	414	100.0
3-4 reports	182	47.5	160	41.8	41	10.7	383	100.0
5+ reports	139	42.8	150	46.2	36	11.1	325	100.0
**3 to <4 years**	**919**	**54**	**620**	**36.4**	**162**	**9.5**	**1,701**	100.0

1 report	508	59.9	272	32.1	68	8.0	848	**100.0**
2 reports	190	50.8	144	38.5	40	10.7	374	100.0
3-4 reports	145	49.0	117	39.5	34	11.5	296	**100.0**
5+ reports	76	41.5	87	47.5	20	10.9	183	**100.0**
**4 years until starting school**	**1,241**	**53.5**	**819**	**35.3**	**260**	**11.2**	**2,320**	100.0
1 report	951	56.1	571	33.7	173	10.2	1,695	100.0
2 reports	178	48.9	136	37.4	50	13.7	364	100.0
3-4 reports	92	46.5	80	40.4	26	13.1	198	100.0
5+ reports	20	31.7	32	50.8	11	17.5	63	**100.0**
**Total**	**57,958**	**71.8**	**18,086**	**22.4**	**4,687**	**5.8**	**80,731**	100.0

AEDC, Australian Early Development Census; NSW, New South Wales. 1. The AEDC DV1 summary indicator was used for the outcome ‘developmental vulnerability on ≥1 AEDC domains’; 2. Medically diagnosed conditions with already identified substantial developmental support needs, as listed in eTable 2; 3. AEDC years include 2009, 2012, 2015, 2018.

eTable8 The risk of developmental vulnerability on ≥1 AEDC domain[1] or medically diagnosed conditions[2], by most serious child protection contact before school, among New South Wales and South Australian children in their first year of full-time school in 2009, 2012, 2015 or 2018. (data table for Figure 1a).

Most serious child protection contact before school	AEDC Year	New South Wales							
		Risk of developmental vulnerability on ≥1 domains or medically diagnosed conditions						Total	
		No		Developmental vulnerability on ≥1 domain		Medically diagnosed conditions			
		n	row %	n	row %	n	row %	n	row %
No contact	2009	61,832	78.1	14,379	18.2	2,962	3.7	79,173	100.0
	2012	66,223	79.0	14,098	16.8	3,534	4.2	83,855	100.0
	2015	68,980	78.3	15,428	17.5	3,708	4.2	88,116	100.0
	2018	71,632	78.8	15,862	17.4	3,414	3.8	90,908	100.0
Report	2009	1,329	65.4	593	29.2	111	5.5	2,033	100.0
	2012	1,589	65.5	676	27.9	162	6.7	2,427	100.0
	2015	1,835	65.4	777	27.7	195	6.9	2,807	100.0
	2018	1,761	64.8	762	28.0	196	7.2	2,719	100.0
Screened-in	2009	5,528	58.9	3,183	33.9	667	7.1	9,378	100.0
	2012	5,982	61.2	3,036	31.1	752	7.7	9,770	100.0
	2015	3,912	61.1	1,952	30.5	543	8.5	6,407	100.0
	2018	3,300	62.3	1,601	30.2	392	7.4	5,293	100.0
Investigated	2009	155	57.4	100	37.0	15	5.6	270	100.0
	2012	148	55.0	94	34.9	27	10.0	269	100.0
	2015	301	58.0	176	33.9	42	8.1	519	100.0
	2018	668	54.5	455	37.1	102	8.3	1,225	100.0
Substantiated	2009	760	48.1	686	43.4	135	8.5	1,581	100.0
	2012	986	49.5	781	39.2	226	11.3	1,993	100.0
	2015	1,096	50.0	853	38.9	242	11.0	2,191	100.0
	2018	1,266	52.8	932	38.9	198	8.3	2,396	100.0
OOHC	2009	540	45.9	447	38.0	189	16.1	1,176	100.0
	2012	681	46.5	520	35.5	263	18.0	1,464	100.0
	2015	644	48.3	463	34.7	227	17.0	1,334	100.0
	2018	706	50.5	489	35.0	203	14.5	1,398	100.0
	**Total**	**301,854**	**75.7**	**78,343**	**19.6**	**18,505**	**4.6**	398,702	100.0
		**South Australia**							
No contact	2009	11,916	75.8	2,972	18.9	825	5.3	15,713	100.0
	2012	12,713	75.3	3,226	19.1	934	5.5	16,873	100.0
	2015	13,378	76.2	3,383	19.3	794	4.5	17,555	100.0
	2018	13,728	76.4	3,484	19.4	761	4.2	17,973	100.0
Report	2009	328	54.5	212	35.2	62	10.3	602	100.0
	2012	476	55.6	293	34.2	87	10.2	856	100.0
	2015	682	58.8	373	32.2	104	9.0	1,159	100.0
	2018	873	58.0	508	33.8	123	8.2	1,504	100.0
Screened-in	2009	375	49.7	298	39.5	82	10.9	755	100.0

	2012	551	49.7	433	39.0	125	11.3	1,109	100.0
	2015	625	51.1	484	39.6	113	9.2	1,222	100.0
	2018	618	49.4	513	41.0	119	9.5	1,250	100.0
Investigated	2009	188	42.5	191	43.2	63	14.3	442	100.0
	2012	213	43.4	223	45.4	55	11.2	491	100.0
	2015	203	46.6	175	40.1	58	13.3	436	100.0
	2018	132	43.6	145	47.9	26	8.6	303	100.0
Substantiated	2009	133	40.8	15	48.8	34	10.4	326	100.0
	2012	155	38.9	189	47.5	54	13.6	398	100.0
	2015	179	40.3	211	47.5	54	12.2	444	100.0
	2018	145	38.6	191	50.8	40	10.6	376	100.0
OOHC	2009	83	38.4	85	39.4	48	22.2	216	100.0
	2012	76	33.6	102	45.1	48	21.2	226	100.0
	2015	94	40.9	99	43.0	37	16.1	230	100.0
	2018	94	34.6	137	50.4	41	15.1	272	100.0
	**Total**	**57,958**	**71.8**	**18,086**	**22.4**	**4,687**	**5.8**	80,731	100.0

AEDC, Australian Early Development Census; NSW, New South Wales. 1. The AEDC DV1 summary indicator was used for the outcome ‘developmental vulnerability on ≥1 AEDC domains’; 2. Medically diagnosed conditions with already identified substantial developmental support needs, as listed in eTable 2.

eTable9 The risk of developmental vulnerability on 1-5 AEDC domains, or medically diagnosed conditions[1], by most serious child protection contact before school, among New South Wales and South Australian children in their first year of full-time school in 2009, 2012, 2015 or 2018. (data table for Figure 1b).

Most serious child protection contact before school	AEDC Year	New South Wales															
		Risk of developmental vulnerability on ≥1 AEDC domains or medically diagnosed conditions														Total	
		No		Developmental vulnerability on 1 domain		Developmental vulnerability on 2 domains		Developmental vulnerability on 3 domains		Developmental vulnerability on 4 domains		Developmental vulnerability on 5 domains		Medically diagnosed conditions			
		n	row %	n	row %	n	row %	n	row %	n	row %	n	row %	n	row %	n	row %
No contact	2009	61,832	78.1	7,881	10.0	3,406	4.3	1,678	2.1	993	1.3	421	0.5	2,962	3.7	79,173	100.0
	2012	66,223	79.0	8,006	9.5	3,245	3.9	1,616	1.9	865	1.0	366	0.4	3,534	4.2	83,855	100.0
	2015	68,980	78.3	8,442	9.6	3,624	4.1	1,916	2.2	999	1.1	447	0.5	3,708	4.2	88,116	100.0
	2018	71,632	78.8	8,487	9.3	3,728	4.1	1,973	2.2	1,129	1.2	545	0.6	3,414	3.8	90,908	100.0
Report	2009	1,329	65.4	270	13.3	153	7.5	88	4.3	49	2.4	33	1.6	111	5.5	2,033	100.0
	2012	1,589	65.5	303	12.5	181	7.5	87	3.6	71	2.9	34	1.4	162	6.7	2,427	100.0
	2015	1,835	65.4	379	13.5	180	6.4	111	4.0	74	2.6	33	1.2	195	6.9	2,807	100.0
	2018	1,761	64.8	361	13.3	191	7.0	99	3.6	70	2.6	41	1.5	196	7.2	2,719	100.0
Screened-in	2009	5,528	58.9	1,362	14.5	817	8.7	493	5.3	327	3.5	184	2.0	667	7.1	9,378	100.0
	2012	5,982	61.2	1,373	14.1	758	7.8	470	4.8	294	3.0	141	1.4	752	7.7	9,770	100.0
	2015	3,912	61.1	822	12.8	496	7.7	316	4.9	212	3.3	106	1.7	543	8.5	6,407	100.0
	2018	3,300	62.3	707	13.4	386	7.3	233	4.4	161	3.0	114	2.2	392	7.4	5,293	100.0
Investigated	2009	155	57.4	33	12.2	23	8.5	20	7.4	11	4.1	13	4.8	15	5.6	270	100.0
	2012	148	55.0	40	14.9	21	7.8	19	7.1	7	2.6	7	2.6	27	10.0	269	100.0
	2015	301	58.0	72	13.9	44	8.5	31	6.0	17	3.3	12	2.3	42	8.1	519	100.0
	2018	668	54.5	186	15.2	110	9.0	79	6.4	43	3.5	37	3.0	102	8.3	1,225	100.0
Substantiated	2009	760	48.1	265	16.8	173	10.9	112	7.1	77	4.9	59	3.7	135	8.5	1,581	100.0
	2012	986	49.5	322	16.2	195	9.8	107	5.4	101	5.1	56	2.8	226	11.3	1,993	100.0
	2015	1,096	50.0	343	15.7	194	8.9	148	6.8	97	4.4	71	3.2	242	11.0	2,191	100.0
	2018	1,266	52.8	372	15.5	210	8.8	153	6.4	115	4.8	82	3.4	198	8.3	2,396	100.0
OOHC	2009	540	45.9	178	15.1	122	10.4	81	6.9	42	3.6	24	2.0	189	16.1	1,176	100.0

	2012	681	46.5	198	13.5	119	8.1	99	6.8	64	4.4	40	2.7	263	18.0	1,464	100.0
	2015	644	48.3	178	13.3	143	10.7	76	5.7	44	3.3	22	1.6	227	17.0	1,334	100.0
	2018	706	50.5	181	12.9	146	10.4	80	5.7	53	3.8	29	2.1	203	14.5	1,398	100.0
	**Total**	**301,854**	**75.7**	**40,761**	**10.2**	**18,665**	**4.7**	**10,085**	**2.5**	**5,915**	**1.5**	**2,917**	**0.7**	**18,505**	**4.6**	**398,702**	**100.0**
		**South Australia**															
No contact	2009	11,916	75.8	1,568	10.0	719	4.6	362	2.3	199	1.3	124	0.8	825	5.3	15,713	100.0
	2012	12,713	75.3	1,683	10.0	788	4.7	412	2.4	230	1.4	113	0.7	934	5.5	16,873	100.0
	2015	13,378	76.2	1,745	9.9	828	4.7	437	2.5	255	1.5	118	0.7	794	4.5	17,555	100.0
	2018	13,728	76.4	1,731	9.6	872	4.9	470	2.6	279	1.6	132	0.7	761	4.2	17,973	100.0
Report	2009	328	54.5	77	12.8	51	8.5	39	6.5	28	4.7	17	2.8	62	10.3	602	100.0
	2012	476	55.6	129	15.1	66	7.7	44	5.1	32	3.7	22	2.6	87	10.2	856	100.0
	2015	682	58.8	161	13.9	95	8.2	50	4.3	42	3.6	25	2.2	104	9.0	1,159	100.0
	2018	873	58.0	201	13.4	127	8.4	95	6.3	46	3.1	39	2.6	123	8.2	1,504	100.0
Screened-in	2009	375	49.7	122	16.2	64	8.5	63	8.3	31	4.1	18	2.4	82	10.9	755	100.0
	2012	551	49.7	174	15.7	96	8.7	80	7.2	51	4.6	32	2.9	125	11.3	1,109	100.0
	2015	625	51.1	186	15.2	115	9.4	80	6.5	62	5.1	41	3.4	113	9.2	1,222	100.0
	2018	618	49.4	181	14.5	122	9.8	90	7.2	71	5.7	49	3.9	119	9.5	1,250	100.0
Investigated	2009	188	42.5	70	15.8	50	11.3	30	6.8	17	3.8	24	5.4	63	14.3	442	100.0
	2012	213	43.4	81	16.5	51	10.4	47	9.6	25	5.1	19	3.9	55	11.2	491	100.0
	2015	203	46.6	64	14.7	45	10.3	22	5.0	28	6.4	16	3.7	58	13.3	436	100.0
	2018	132	43.6	46	15.2	39	12.9	30	9.9	12	4.0	18	5.9	26	8.6	303	100.0
Substantiated	2009	133	40.8	54	16.6	35	10.7	23	7.1	30	9.2	17	5.2	34	10.4	326	100.0
	2012	155	38.9	64	16.1	43	10.8	33	8.3	34	8.5	15	3.8	54	13.6	398	100.0
	2015	179	40.3	65	14.6	57	12.8	41	9.2	28	6.3	20	4.5	54	12.2	444	100.0
	2018	145	38.6	54	14.4	43	11.4	37	9.8	38	10.1	19	5.1	40	10.6	376	100.0
OOHC	2009	83	38.4	30	13.9	16	7.4	20	9.3	10	4.6	9	4.2	48	22.2	216	100.0
	2012	76	33.6	33	14.6	28	12.4	17	7.5	11	4.9	13	5.8	48	21.2	226	100.0
	2015	94	40.9	38	16.5	21	9.1	22	9.6	11	4.8	7	3.0	37	16.1	230	100.0
	2018	94	34.6	48	17.6	40	14.7	16	5.9	21	7.7	12	4.4	41	15.1	272	100.0
	**Total**	**57,958**	**71.8**	**8,605**	**10.7**	**4,411**	**5.5**	**2,560**	**3.2**	**1,591**	**2.0**	**919**	**1.1**	**4,687**	**5.8**	**80,731**	**100.0**

AEDC, Australian Early Development Census; OOHC, out-of-home care. 1. Medically diagnosed conditions with already identified substantial developmental support needs, as listed in eTable 2.

eTable10 The risk of developmental vulnerability on ≥1 domain[1] or medically diagnosed conditions[2] at age five, by age at first child protection contact before school, among New South Wales and South Australian children in their first year of school (all AEDC years combined[3]) (data table for Figure 2).

Age at first child protection system contact	New South Wales: Risk of developmental vulnerability on ≥1 domain or medically diagnosed conditions						Total	
	No		Developmental vulnerability on ≥1 domain		Diagnosed conditions			
	n	row %	n	row %	n	row %	n	row %
No contact	268,667	78.5	59,767	17.5	13,618	4.0	342,052	100.0
< 1 year	12,242	54.8	7,976	35.7	2,102	9.4	22,320	100.0
1 to < 2 years	5,914	59.5	3,272	32.9	759	7.6	9,945	100.0
2 to <3 years	5,082	60.7	2,625	31.3	668	8.0	8,375	100.0
3 to <4 years	4,492	61.4	2,215	30.3	610	8.3	7,317	100.0
4 years until starting school	5,457	62.8	2,488	28.6	748	8.6	8,693	100.0
**Total**	**301,854**	**75.7**	**78,343**	**19.6**	**18,505**	**4.6**	**398,702**	**100.0**
	**South Australia**							
No contact	51,735	76.0	13,065	19.2	3,314	4.9	68,114	100.0
< 1 year	1,982	44.5	1,926	43.2	549	12.3	4,457	100.0
1 to < 2 years	1,058	48.4	922	42.1	208	9.5	2,188	100.0
2 to <3 years	1,023	52.4	734	37.6	194	9.9	1,951	100.0
3 to <4 years	919	54.0	620	36.4	162	9.5	1,701	100.0
4 years until starting school	1,241	53.5	819	35.3	260	11.2	2,320	100.0
**Total**	**57,958**	**71.8**	**18,086**	**22.4**	**4,687**	**5.8**	**80,731**	**100.0**

AEDC, Australian Early Development Census. 1. The AEDC DV1 summary indicator was used for the outcome 'developmental vulnerability on ≥1 AEDC domains'; 2. Medically diagnosed conditions with already identified substantial developmental support needs, as listed in eTable 2; 3. 2009, 2012, 2015, and 2018 AEDC cycles.

eTable11 The risk of developmental vulnerability on ≥1 domain[1] or medically diagnosed conditions[2] at age five, by number of child protection reports before school, among New South Wales and South Australian children in their first year of school in 2009, 2012, 2015 or 2018 (data table for Figure 3).

Number of reports before starting school	AEDC Year	New South Wales: Risk of developmental vulnerability on ≥1 domain or medically diagnosed conditions						Total	
		No		Developmental vulnerability on ≥1 domain		Diagnosed conditions			
		n	row %	n	row %	n	row %	n	row %
0 Reports	2009	61,860	78.1	14,388	18.2	2,968	3.7	79,216	100.0
	2012	66,228	79.0	14,101	16.8	3,536	4.2	83,865	100.0
	2015	68,984	78.3	15,429	17.5	3,710	4.2	88,123	100.0
	2018	71,642	78.8	15,863	17.4	3,417	3.8	90,922	100.0
1 Report	2009	3,258	64.1	1,504	29.6	317	6.2	5,079	100.0
	2012	3,573	66.5	1,412	26.3	385	7.2	5,370	100.0
	2015	3,074	66.7	1,214	26.3	321	7.0	4,609	100.0
	2018	2,977	66.0	1,229	27.2	308	6.8	4,514	100.0
2 Reports	2009	1,510	60.5	817	32.7	168	6.7	2,495	100.0
	2012	1,600	61.8	782	30.2	208	8.0	2,590	100.0
	2015	1,280	60.3	659	31.0	184	8.7	2,123	100.0
	2018	1,270	61.9	620	30.2	162	7.9	2,052	100.0
3-4 Reports	2009	1,350	55.9	876	36.3	189	7.8	2,415	100.0
	2012	1,538	58.5	889	33.8	203	7.7	2,630	100.0
	2015	1,283	57.6	716	32.2	227	10.2	2,226	100.0
	2018	1,214	57.6	722	34.3	172	8.2	2,108	100.0
5+ Reports	2009	2,166	49.2	1,803	40.9	437	9.9	4,406	100.0
	2012	2,670	50.2	2,021	38.0	632	11.9	5,323	100.0
	2015	2,147	50.0	1,631	38.0	515	12.0	4,293	100.0
	2018	2,230	51.3	1,667	38.4	446	10.3	4,343	100.0
	**Total**	**301,854**	**75.7**	**78,343**	**19.6**	**18,505**	**4.6**	**398,702**	**100.0**
		**South Australia**							
0 Reports	2009	11,926	75.8	2,978	18.9	830	5.3	15,734	100.0
	2012	12,725	75.3	3,231	19.1	941	5.6	16,897	100.0
	2015	13,385	76.2	3,386	19.3	797	4.5	17,568	100.0
	2018	13,730	76.4	3,485	19.4	761	4.2	17,976	100.0
1 Report	2009	534	54.8	335	34.4	105	10.8	974	100.0
	2012	725	55.2	453	34.5	136	10.4	1,314	100.0
	2015	857	59.3	471	32.6	116	8.0	1,444	100.0
	2018	771	58.9	438	33.5	100	7.6	1,309	100.0
2 Reports	2009	203	48.7	167	40.0	47	11.3	417	100.0
	2012	259	49.5	207	39.6	57	10.9	523	100.0
	2015	314	51.1	236	38.4	65	10.6	615	100.0
	2018	356	57.1	204	32.7	63	10.1	623	100.0
3-4 Reports	2009	176	42.0	192	45.8	51	12.2	419	100.0
	2012	242	44.7	239	44.2	60	11.1	541	100.0

	2015	288	49.8	223	38.6	67	11.6	578	100.0
	2018	315	47.7	280	42.4	65	9.8	660	100.0
5+ Reports	2009	184	36.1	245	48.0	81	15.9	510	100.0
	2012	233	34.4	336	49.6	109	16.1	678	100.0
	2015	317	37.7	409	48.6	115	13.7	841	100.0
	2018	418	37.7	571	51.4	121	10.9	1,110	100.0
	**Total**	**57,958**	**71.8**	**18,086**	**22.4**	**4,687**	**5.8**	**80,731**	**100.0**

AEDC, Australian Early Development Census; NSW, New South Wales. 1. The AEDC DV1 summary indicator was used for the outcome 'developmental vulnerability on ≥1 AEDC domains'; 2. Medically diagnosed conditions with already identified substantial developmental support needs, as listed in eTable 2.

eTable12 The risk of developmental vulnerability on ≥1 domain[1] or medically diagnosed conditions[2] at age five, by most serious child protection contact and number of child protection reports before school, among New South Wales and South Australian children in their first year of school (all AEDC years combined[3]). (data table for Figure 4).

Most serious child protection contact before school	Number of reports before starting school	New South Wales: Risk of developmental vulnerability on ≥1 domain or medically diagnosed conditions						Total	
		No		Developmental vulnerability on ≥1 domain		Diagnosed conditions			
		n	row %	n	row %	n	row %	n	row %
No contact	No report	268,667	78.5	59,767	17.5	13,618	4.0	342,052	100.0
Report	1 Report	5,124	66.3	2,130	27.5	480	6.2	7,734	100.0
	2 Reports	944	62.2	461	30.4	113	7.4	1,518	100.0
	3-4 Reports	370	61.0	182	30.0	55	9.1	607	100.0
	5+ Reports	76	59.8	35	27.6	16	12.6	127	100.0
Screened-in	1 Report	6,957	65.8	2,871	27.2	744	7.0	10,572	100.0
	2 Reports	3,960	61.3	2,017	31.2	486	7.5	6,463	100.0
	3-4 Reports	3,808	58.4	2,205	33.8	502	7.7	6,515	100.0
	5+ Reports	3,997	54.8	2,679	36.7	622	8.5	7,298	100.0
Investigated	1 Report	248	63.1	118	30.0	27	6.9	393	100.0
	2 Reports	220	57.7	132	34.6	29	7.6	381	100.0
	3-4 Reports	246	52.0	191	40.4	36	7.6	473	100.0
	5+ Reports	558	53.9	384	37.1	94	9.1	1,036	100.0
Substantiated	1 Report	469	63.7	211	28.7	56	7.6	736	100.0
	2 Reports	430	61.3	204	29.1	67	9.6	701	100.0
	3-4 Reports	718	55.1	467	35.8	119	9.1	1,304	100.0
	5+ Reports	2,491	46.0	2,370	43.7	559	10.3	5,420	100.0
OOHC	No report	47	63.5	14	18.9	13	17.6	74	100.0
	1 Report	84	61.3	29	21.2	24	17.5	137	100.0
	2 Reports	106	53.8	64	32.5	27	13.7	197	100.0
	3-4 Reports	243	50.6	158	32.9	79	16.5	480	100.0
	5+ Reports	2,091	46.6	1,654	36.9	739	16.5	4,484	100.0
	**Total**	**301,854**	**75.7**	**78,343**	**19.6**	**18,505**	**4.6**	**398,702**	**100.0**
		**South Australia**							
No contact	No report	51,735	76.0	13,065	19.2	3,314	4.9	68,114	100.0
Report	1 Report	1,699	58.3	964	33.1	249	8.6	2,912	100.0
	2 Reports	416	55.8	245	32.9	84	11.3	745	100.0
	3-4 Reports	197	53.2	137	37.0	36	9.7	370	100.0
	5+ Reports	47	50.0	40	42.6	7	7.4	94	100.0
Screened-in	1 Report	874	57.6	507	33.4	136	9.0	1,517	100.0
	2 Reports	454	50.5	350	38.9	95	10.6	899	100.0
	3-4 Reports	479	47.1	433	42.5	106	10.4	1,018	100.0
	5+ Reports	362	40.1	438	48.6	102	11.3	902	100.0
Investigated	1 Report	198	50.4	155	39.4	40	10.2	393	100.0
	2 Reports	134	49.6	108	40.0	28	10.4	270	100.0

	3-4 Reports	163	46.2	153	43.3	37	10.5	353	100.0
	5+ Reports	241	36.7	318	48.5	97	14.8	656	100.0
Substantiated	1 Report	91	59.9	49	32.2	12	7.9	152	100.0
	2 Reports	91	50.0	76	41.8	15	8.2	182	100.0
	3-4 Reports	126	43.2	135	46.2	31	10.6	292	100.0
	5+ Reports	304	33.1	490	53.4	124	13.5	918	100.0
OOHC	No report	31	50.8	15	24.6	15	24.6	61	100.0
	1 Report	25	37.3	22	32.8	20	29.9	67	100.0
	2 Reports	37	45.1	35	42.7	10	12.2	82	100.0
	3-4 Reports	56	33.9	76	46.1	33	20.0	165	100.0
	5+ Reports	198	34.8	275	48.3	96	16.9	569	100.0
	**Total**	**57,958**	**71.8**	**18,086**	**22.4**	**4,687**	**5.8**	**80,731**	**100.0**

AEDC, Australian Early Development Census; NSW, New South Wales; OOHC, out-of-home care. 1. The AEDC DV1 summary indicator was used for the outcome 'developmental vulnerability on ≥1 AEDC domains'; 2. Medically diagnosed conditions with already identified substantial developmental support needs, as listed in eTable 2; 3. 2009, 2012, 2015, and 2018 AEDC cycles.

eTable13 The number and percent of children with the most common combinations of number and type of developmentally vulnerable domains among New South Wales and South Australian children with valid AEDC domain outcomes[1] (combined for all AEDC years[2]), according to their most serious level of child protection contact by their first year of full-time school. The percent scale (right-hand Y-axis) is comparable across the jurisdictions. (data table for Figure 5).

Developmentally vulnerable on the following domain:					New South Wales – Most serious child protection contact before school							
Physical health and wellbeing	Social competence	Emotional maturity	Language and cognitive skills (school-based)	Communication skills and general knowledge	No contact		Reports including investigated		Substantiated		OOHC	
					n	%	n	%	n	%	n	%
No	No	No	No	No	268,667	82.0	26,508	66.7	4,108	56.1	2,571	57.5
Yes	No	No	No	No	9,103	2.8	1,986	5.0	517	7.1	198	4.4
No	Yes	No	No	No	5,836	1.8	989	2.5	166	2.3	154	3.4
No	No	Yes	No	No	5,512	1.7	904	2.3	176	2.4	168	3.8
No	No	No	Yes	No	3,322	1.0	875	2.2	225	3.1	109	2.4
No	No	No	No	Yes	8,600	2.6	1,094	2.8	207	2.8	103	2.3
Yes	Yes	No	No	No	1,421	0.4	351	0.9	80	1.1	45	1.0
Yes	No	Yes	No	No	770	0.2	197	0.5	46	0.6	32	0.7
Yes	No	No	Yes	No	734	0.2	300	0.8	99	1.4	46	1.0
Yes	No	No	No	Yes	2,185	0.7	464	1.2	124	1.7	51	1.1
No	Yes	Yes	No	No	4,157	1.3	1,036	2.6	196	2.7	266	5.9
No	Yes	No	Yes	No	504	0.2	150	0.4	43	0.6	19	0.4
No	Yes	No	No	Yes	1,682	0.5	261	0.7	49	0.7	25	0.6
No	No	Yes	Yes	No	230	0.1	70	0.2	22	0.3	7	0.2
No	No	Yes	No	Yes	455	0.1	68	0.2	12	0.2	-	-
No	No	No	Yes	Yes	1,677	0.5	414	1.0	91	1.2	30	0.7
Yes	Yes	Yes	No	No	1,573	0.5	497	1.2	139	1.9	97	2.2
Yes	Yes	No	Yes	No	323	0.1	129	0.3	31	0.4	23	0.5
Yes	Yes	No	No	Yes	1,210	0.4	269	0.7	55	0.8	23	0.5
Yes	No	Yes	Yes	No	64	0.0	42	0.1	15	0.2	-	-
Yes	No	Yes	No	Yes	233	0.1	53	0.1	14	0.2	9	0.2
Yes	No	No	Yes	Yes	893	0.3	348	0.9	108	1.5	37	0.8

No	Yes	Yes	Yes	No	590	0.2	200	0.5	50	0.7	63	1.4
No	Yes	Yes	No	Yes	1,140	0.3	234	0.6	49	0.7	38	0.8
No	Yes	No	Yes	Yes	933	0.3	221	0.6	45	0.6	34	0.8
No	No	Yes	Yes	Yes	129	0.0	32	0.1	6	0.1	-	-
Yes	Yes	Yes	Yes	No	424	0.1	207	0.5	83	1.1	40	0.9
No	Yes	Yes	Yes	Yes	802	0.2	263	0.7	64	0.9	34	0.8
Yes	No	Yes	Yes	Yes	103	0.0	44	0.1	19	0.3	6	0.1
Yes	Yes	No	Yes	Yes	1,301	0.4	446	1.1	128	1.7	54	1.2
Yes	Yes	Yes	No	Yes	1,309	0.4	360	0.9	94	1.3	67	1.5
Yes	Yes	Yes	Yes	Yes	1,779	0.5	755	1.9	268	3.7	115	2.6
					**327,661**	**100**	**39,767**	**100**	**7,329**	**100**	**4,475**	**100**
					**South Australia**							
**Developmentally vulnerable on the following domain:**					**Most serious child protection contact before school**							
**Physical health and wellbeing**	**Social competence**	**Emotional maturity**	**Language and cognitive skills (school-based)**	**Communication skills and general knowledge**	**No contact**		**Reports including those investigated**		**Substantiated**		**OOHC**	
					**n**	**%**	**n**	**%**	**n**	**%**	**n**	**%**
No	No	No	No	No	51,735	80.0	5,264	58.1	612	45.4	347	45.3
Yes	No	No	No	No	1,959	3.0	531	5.9	102	7.6	38	5.0
No	Yes	No	No	No	1,049	1.6	235	2.6	25	1.9	33	4.3
No	No	Yes	No	No	1,512	2.3	267	3.0	45	3.3	34	4.4
No	No	No	Yes	No	810	1.3	254	2.8	37	2.7	23	3.0
No	No	No	No	Yes	1,305	2.0	181	2.0	26	1.9	20	2.6
Yes	Yes	No	No	No	312	0.5	95	1.1	20	1.5	12	1.6
Yes	No	Yes	No	No	218	0.3	86	1.0	15	1.1	8	1.0
Yes	No	No	Yes	No	188	0.3	88	1.0	25	1.9	-	-
Yes	No	No	No	Yes	375	0.6	104	1.2	26	1.9	8	1.0
No	Yes	Yes	No	No	1,190	1.8	317	3.5	45	3.3	58	7.6
No	Yes	No	Yes	No	129	0.2	39	0.4	5	0.4	-	-
No	Yes	No	No	Yes	227	0.4	49	0.5	6	0.4	-	-
No	No	Yes	Yes	No	77	0.1	23	0.3	<5	<0.5	-	-

No	No	Yes	No	Yes	116	0.2	18	0.2	<5	<0.5		
No	No	No	Yes	Yes	341	0.5	89	1.0	27	2.0	6	0.8
Yes	Yes	Yes	No	No	474	0.7	202	2.2	33	2.5	34	4.4
Yes	Yes	No	Yes	No	77	0.1	50	0.6	11	0.8	5	0.7
Yes	Yes	No	No	Yes	208	0.3	60	0.7	9	0.7	-	-
Yes	No	Yes	Yes	No	27	0.0	11	0.1	5	0.4	-	-
Yes	No	Yes	No	Yes	54	0.1	25	0.3	<5	<0.5	-	-
Yes	No	No	Yes	Yes	200	0.3	94	1.0	24	1.8	7	0.9
No	Yes	Yes	Yes	No	175	0.3	92	1.0	22	1.6	11	1.4
No	Yes	Yes	No	Yes	250	0.4	56	0.6	11	0.8	-	-
No	Yes	No	Yes	Yes	158	0.2	59	0.7	12	0.9	6	0.8
No	No	Yes	Yes	Yes	40	0.1	13	0.1	<5	<0.5	-	-
Yes	Yes	Yes	Yes	No	147	0.2	114	1.3	30	2.2	17	2.2
No	Yes	Yes	Yes	Yes	199	0.3	87	1.0	21	1.6	14	1.8
Yes	No	Yes	Yes	Yes	30	0.1	16	0.2	5	0.4	-	-
Yes	Yes	No	Yes	Yes	265	0.4	118	1.3	38	2.8	-	-
Yes	Yes	Yes	No	Yes	312	0.5	105	1.2	32	2.4	17	2.2
Yes	Yes	Yes	Yes	Yes	487	0.8	320	3.5	71	5.3	41	5.4
					**64,646**	**100**	**9,062**	**100**	**1,349**	**100**	**766**	**100**

1. Non-missing data for the AEDC domain indicators (i.e. ‘developmentally vulnerable’ on each of the five AEDC domains); 2. 2009, 2012, 2015, and 2018 AEDC cycles.
